# A comparison of the statistical performance of different meta-analysis models for the synthesis of subgroup effects from randomized clinical trials

**DOI:** 10.1186/s12874-019-0831-8

**Published:** 2019-10-26

**Authors:** Bruno R. da Costa, Alex J. Sutton

**Affiliations:** 1 Applied Health Research Center (AHRC), Li Ka Shing Knowledge Institute of St. Michael’s Hospital, Toronto, Canada; 2 Institute of Health Policy Management and Evaluation, University of Toronto, Toronto, Canada; 3 Institute of Primary Health Care (BIHAM), University of Bern, Bern, Switzerland; 4 Department of Health Sciences, University of Leicester, Leicester, UK

**Keywords:** Individual patient data, Meta-analysis, Random-effects, Evidence synthesis, Interaction effects, Subgroup analysis

## Abstract

**Background:**

When investigating subgroup effects in meta-analysis, it is unclear whether accounting in meta-regression for between-trial variation in treatment effects, but not between-trial variation in treatment interaction effects when such effects are present, leads to biased estimates, coverage problems, or wrong standard errors, and whether the use of aggregate data (AD) or individual-patient-data (IPD) influences this assessment.

**Methods:**

Seven different models were compared in a simulation study. Models differed regarding the use of AD or IPD, whether they accounted for between-trial variation in interaction effects, and whether they minimized the risk of ecological fallacy.

**Results:**

Models that used IPD and that allowed for between-trial variation of the interaction effect had less bias, better coverage, and more accurate standard errors than models that used AD or ignored this variation. The main factor influencing the performance of models was whether they used IPD or AD. The model that used AD had a considerably worse performance than all models that used IPD, especially when a low number of trials was included in the analysis.

**Conclusions:**

The results indicate that IPD models that allow for the between-trial variation in interaction effects should be given preference whenever investigating subgroup effects within a meta-analysis.

## Background

Meta-analysis is an essential tool for evidence-based clinical practice [1]. It allows the combination of treatment effect estimates across two or more trials, which has two main advantages. First, it provides a summary treatment effect estimate for patient, clinicians, or policy-makers seeking information about the effectiveness of a treatment. Because these stakeholders are commonly faced with a large number of scientific literature publications on which they need to base their decisions, a summary treatment effect estimate(s) is(are) (or summary distribution of treatment effects, in the presence of heterogeneity when random effect models are used) likely to facilitate the decision-making process. Second, it provides a more precise treatment effect estimate, that is, a treatment effect with narrower confidence intervals, minimizing sampling error. Randomized controlled trials (RCTs) are considered the gold-standard study design to investigate the effectiveness of treatment interventions [2]. Meta-analyses of randomized controlled trials offer, therefore, the optimal basis for evidence-based clinical practice.

The data used to conduct a meta-analysis can be of two forms: an average treatment effect from each study, such as a difference in means for continuous outcomes or a risk ratio for binary outcomes, or it can be at the patient level, where the outcome of interest is available individually for each of the patients included in the trial [3]. The vast majority of meta-analyses are based on average treatment effects, which are commonly referred to as aggregate data (AD) meta-analyses, while a small proportion use individual patient data (IPD) or a combination of AD and IPD [3, 4]. The reason why most meta-analyses use AD is because they are readily available in published, publicly available reports of RCTs. Getting access to IPD is usually difficult or even impossible, as in most cases IPD are not publicly available, clinical trialists often choose not to share them, or datasets have been lost or misplaced [5]. However, the use of IPD in meta-analysis offers significant statistical advantages, such as more precise results with narrower confidence intervals in some scenarios or the proper investigation of subgroup treatment effects [6].

Investigation of subgroup effects is an important part of clinical research [7]. While a treatment may be of low clinical relevance in the broader population of patients with a particular disease, we may identify subgroups of patients who may significantly benefit from this treatment. An intuitive example would be weight reduction in patients with knee osteoarthritis (OA) [8]. While weight reduction may have a significant effect on the knee pain of OA patients who are overweight, it would not work or even be detrimental for those patients with normal or below normal body weight. The investigation of the source of variation of treatment effects between patients is therefore of crucial relevance for the use of trial findings in the clinical setting. Otherwise, useful treatments may be discarded solely based on its irrelevant effect at the overall patient population or we may fail to identify subgroups of patients who are more likely to respond to a treatment that only has a negligible effect in the overall patient population of interest.

Subgroup analyses in single trials are often underpowered since RCTs are generally only powered to identify a treatment effect at the overall patient population. Because of this, meta-analyses that combine data from several trials become a powerful tool in conducting subgroup analyses that have enough statistical power to identify minimal clinically relevant treatment effects in specific subgroups of patients. The investigation of subgroup effects within a meta-analysis can be conducted using meta-regression. Regression analyses can be used to explain the variation of treatment effects across different trials [9]. Both patient and trial level characteristics can be used in these regression analyses. While the investigation of the influence of trial level characteristics, such as methodological quality, in the variation of treatment effects can be done using either AD or IPD, the investigation of patient level characteristics, such as age or gender, using only AD may be biased due to ecological fallacy (also known as aggregation bias) since average patient characteristics are regressed against average trial outcomes [10]. Thus, regression analyses using IPD is considered the gold standard when investigating subgroup effects of patient-level characteristics in a meta-analysis since individual patient’s characteristics can be regressed against the individual’s outcome.

In meta-analysis, it is usually considered important to take into account the between-trial variation in the effect of interest that is being pooled across trials. For instance, when meta-analyzing treatment effects across trials, we may notice a considerable between-trial variation. This indicates that there is variability around the overall treatment effect estimate, which may be caused by more than just chance (i.e. sampling error). When this is the case, it is important to use methods that incorporate this additional variability in the estimation of confidence intervals around the pooled effect estimate. This is done in meta-analysis by using a random-effects approach, which accounts for the between-trial variation in the treatment effect [11, 12] and allows for the distribution of effects to be quantified [13]. Likewise, it may be important to take into account the between-trial variation in the interaction effect between patient-level characteristics and treatment effect, when using regression analyses to conduct subgroup analyses in a meta-analyses. However, regression models implemented in statistical software usually account only for between-trial variation in the treatment effect when pooling interaction effects across trials. It is unclear whether accounting for between-trial variance only in treatment effects, but not in interaction effects, leads to biased estimates, coverage problems, or under or overestimation of standard errors, when estimating overall mean effects, and whether this could be influenced by the use of AD or IPD data for the analysis.

Thus, the main purpose of this simulation study is to understand whether the current approach for combining interaction effects, which accounts only for between-trial variation in treatment effects, but ignores the variation of interaction effects across trials, leads to a suboptimal estimation of a pooled interaction effect across trials. In Methods section, the methods used in the current simulation study, including a description of the statistical models and performance measures used in this investigation will be described. In Results section, the results of the simulation analyses will be presented. In Discussion section, the results will be discussed in the context of previous investigations; limitations and strengths will be described, and conclusions will be presented.

## Methods

We now describe i) the methods used to generate the simulated datasets used for the performance analysis; ii) the analysis models that will be compared; and iii) the performance measures that will be used to compare these models. The reporting of the methods and results of this simulation study follow the guidelines proposed by Morris et al. and Burton et al. [14, 15].

### Data-generating mechanisms

#### Linear predictor used for data generation

A framework was developed to investigate how different models for the assessment of treatment interaction effects in meta-analyses with continuous outcomes perform. Thus, we simulated datasets in order to assess the performance of different models to quantify the interaction between treatment effect and patients’ age within an IPD meta-analysis framework. We assume a linear association between age and knee OA pain, and a linear association between age and the treatment effect. The simulated data mimicked trials of high dose Naproxen compared with placebo for the treatment of knee OA pain quantified with a single assessment at the end of treatment using a 10-cm visual analogue scale (VAS), where 0 means no pain and 10 means the worst imaginable pain [16].

The following linear predictor was used to simulate this dataset (Table 1):

4.1 $$ {\displaystyle \begin{array}{c}{y}_{ij}=6.0+\left({treat}_{ij}\ast beta.{treat}_i\right)+\left({age}_{ij}\ast 0.1\right)+\left({treat}_{ij}\ast {age}_{ij}\ast beta.{inter}_i\right)+{u}_{ij}+{v}_{ij}\ast {treat}_{ij}+{\varepsilon}_{ij}\\ {}{u}_{ij}\sim N\left(0,{\sigma}^2\right),\kern1em {v}_{ij}\sim N\left(0,{\tau}^2\right),\kern0.5em \mathit{\operatorname{cov}}\left({u}_{ij},{v}_{ij}\right)=\rho \sigma \tau \\ {}{\varepsilon}_{ij}\sim N\left(0, res.{\mathit{\operatorname{var}}}_i\right)\\ {}\  beta.{treat}_i\sim N\left(-1.0,{V}_1\right)\\ {}\  beta.{inter}_i\sim N\left(\delta, {V}_2\right)\\ {}\ {age}_{ij}\sim N\left( mean.{age}_i, variance.{age}_i\right)\\ {}\  mean.{age}_i\sim N\left(67,20.25\right)\\ {}\  variance.{age}_i\sim N\left(49,1\right)\end{array}} $$

where *y*_*ij*_ is the predicted knee OA pain measured on a 0 to 10 VAS^1^ at the end of treatment on the *j*^th^ patient in the *i*^th^ trial, *treat*_*ij*_ and *age*_*ij*_ are, respectively, the treatment received (a binary indicator variable coded 0 for the control group and 1 for the Naproxen group) and age of the *j*^th^ patient in the *i*^th^ trial, *beta.treat*_*i*_ is the between-group difference in mean VAS (i.e. treatment effect) in trial *i* and comes from a normal distribution with mean − 1 and variance *V*_*1*,_ *beta.inter*_*i*_ is the interaction between the treatment effect and age in trial *i*, *u*_*ij*_ and *v*_*ij*_ are, respectively, the random intercept and the random slope of the treatment effect on the *j*^th^ patient in the *i*^th^ trial, and res.var. is the residual variance of knee OA pain in the *i*^th^ trial. *age* is a continuous variable, with a mean that was drawn from a normal distribution with mean of 67 and variance of 20.25, and a variance was drawn from a normal distribution with mean 49 and variance of 1, which allowed the variation of mean.age and variance.age across simulated trials. *beta.inter* comes from a normal distribution with mean *δ* and variance *V*_*2*_. *δ* was set as − 0.01 for the main analysis and as 0 for a sensitivity analysis to assess model performance when there is actually no interaction effect. The *δ* of − 0.01 assumes that the treatment effect improves by − 0.01 (as a negative effect means a larger treatment effect in this case) by every increase of 1 year in age. *V*_*2*_ was set as 0.05 (high but plausible heterogeneity) for the main analysis and as 0.5 (implausible high heterogeneity), 0.005 (low heterogeneity) or 0 (no heterogeneity) for sensitivity analyses. *u*_*ij*_ and *v*_*ij*_ are assumed to follow a multivariate normal distribution with mean 0, a variance–covariance matrix ∑, and correlation *ρ* of 0.8. The variance of the random intercept represented by *σ*^2^ and the variance of the random slope of the treatment effect by *τ*^2^ and they have a correlation *ρ*. Thus, this model simulates heterogeneity both in treatment effects and in interaction effects, but the latter is usually not modelled in meta-analysis.

**Table 1:** Varying and fixed parameters of simulated datasets that were used to compare the performance measures of different models

Parameters	Value in simulation	Statistical notation in the linear predictor used for simulation of dataset^b^
*Varying-parameters*		
Number of trials	6, 10, 16, 20, 26, 30, 40, 50	–
Magnitude of the interaction effect^a^	0, −0.01	*beta.inter*
Between-trial variance of the interaction effect^a^	0, 0.005, 0.05, 0.5	*V*_*2*_
*Fixed-parameters*		
Number of patients included in each trial	between 30 and 199 patients in small trials, and 200 and 400 patients in large trials	*j*
Ratio of small to large trials	1:1	*–*
Number of trial arms	2	*treat*
Ratio of randomization	1:1	–
Magnitude of the treatment effect^a^	−1	*beta.treat*
Between-trial variance of the treatment effect^a^	0.063	*V*_*1*_
Effect of age on the outcome	0.05	*age*
Random intercept variance	0.25	*σ* ^2^
Random slope variance	0.04	*τ* ^2^
Correlation between random intercept and random slope variances	0.8	*ρ*
Age of individual patients	*N*(*mean*. *age*, *variance*. *age*)	*age*
Mean age of patients within trials	*N*(67,20.25)	*mean*. *age*
Variance of age within trials	*N*(49, 1)	*variance*. *age*

^a^On a 0 to 10 visual analogue scale

^b^See Eq. 4.1

#### Varying parameters for data generation

It was of interest to assess the performance of the models under different scenarios commonly relevant to evidence synthesis. Of main interest was to see how model performance was dependent on: the number of trials included in the meta-analysis; the magnitude of the interaction effect between treatment effect and age; and the magnitude of the between-trial heterogeneity of the interaction effect (Table 1). Our estimand *θ*, the interaction effect, was assumed to be − 0.01 or 0 on a 0 to 10 VAS. An interaction effect of − 0.01 means that for every 10 years increase in patients’ age, the treatment effect comparing Naproxen and placebo will increase by − 0.1, which means that Naproxen will have a linear increase in its effect as compared to placebo as patients get older. This is based on an assumption that older patients will have more pain at baseline, and that treatment effects are directly proportional to pain at baseline. A null interaction effect (i.e. 0) assumes that the treatment effect does not vary according to patients’ age.

Thus, model performance was assessed in 64 different simulated scenarios (Table 2). These datasets had 6, 10, 16, 20, 26, 30, 40, or 50 trials, an interaction effect between the treatment effect and age of − 0.01 or 0, and a between-trial variance of the interaction effect of 0 (no heterogeneity), 0.005 (low heterogeneity), 0.05 (high but plausible heterogeneity), or 0.5 (implausible high heterogeneity). All simulated datasets had an even number of trials, so that datasets could be equally divided into trials with a low or large number of patients included, as explained below.

**Table 2:** Simulated scenarios used to compare the performance measures of different models.

Interaction effect			
-0.01		0	
Number of trials	Between-trial variance in the interaction effect	Number of trials	Between-trial variance in the interaction effect
6	0	6	0
10	0	10	0
16	0	16	0
20	0	20	0
26	0	26	0
30	0	30	0
40	0	40	0
50	0	50	0
6	0.005	6	0.005
10	0.005	10	0.005
16	0.005	16	0.005
20	0.005	20	0.005
26	0.005	26	0.005
30	0.005	30	0.005
40	0.005	40	0.005
50	0.005	50	0.005
6	0.05	6	0.05
10	0.05	10	0.05
16	0.05	16	0.05
20	0.05	20	0.05
26	0.05	26	0.05
30	0.05	30	0.05
40	0.05	40	0.05
50	0.05	50	0.05
6	0.5	6	0.5
10	0.5	10	0.5
16	0.5	16	0.5
20	0.5	20	0.5
26	0.5	26	0.5
30	0.5	30	0.5
40	0.5	40	0.5
50	0.5	50	0.5

#### Fixed parameters for data generation

The simulation scenarios described above all shared the same fixed parameters (Table 1) regarding: number of patients included in each trial; number of trial arms; the ratio of randomization; the magnitude of the treatment effect; the between-trial variance of the treatment effect; and the effect of age on knee OA pain. Each of these is described in turn below.

Number of patients included in the trial: half of the trials in the simulated dataset were small and half were large. Trials were considered large if they had on average at least 100 patients in each arm [17]. The total number of patients included in each trial varied randomly between 30 and 199 patients in small trials, and 200 and 400 patients in large trials.

Number of trial arms: all trials had two arms.

Ratio of randomization: all trials had a 1:1 randomization.

Magnitude of the treatment effect: the magnitude of the treatment effect was an effect size of − 1 on a 0 to 10 VAS, favoring patients that received high dose Naproxen as compared to those who received placebo (i.e. lower pain on average among patients that received Naproxen than those that received placebo). Assuming a standard deviation of 2.5, this corresponds to a small to moderate, and minimally clinically relevant effect of − 0.4 standard deviation units, which was the reported effect of high dose Naproxen as compared to placebo in a recently published network meta-analysis of RCTs comparing different types of non-steroidal anti-inflammatory drugs to placebo for the treatment of patients with knee OA [16].

Between-trial variance of the treatment effect: we used a moderate between-trial variance of 0.063. This assumes that the treatment effect comes from a normal distribution with approximately 95% of the trial effects ranging between − 1.5, which is considered a moderate to large treatment effect in knee OA in favour of Naproxen, to 0.5, indicating a small, clinically irrelevant treatment effect in favour of placebo.

Effect of age on knee OA pain: it was assumed that the slope of age in knee OA pain would correspond to a yearly increase in pain of 0.05 on a 0 to 10 VAS.

#### Software used to generate simulated data

Data were simulated in Stata 14.2 using the 64-bit Mersenne twister for random number generation. The input seed was ‘1234’.

#### Code for data generation

The code used for data generation is presented in Additional file 1: Appendix A to facilitate reproducibility and understanding of the methods used in this simulation study.

### Estimand

The estimand *θ* is the interaction effect between the treatment effect and age, which is described in the models below as *beta.interC*. That is, the magnitude by which the treatment effect changes for every unit increase in age.

### Models assessed

The performance of seven different models were compared. Models 1, 2, and 3 were based on the proposed model by Riley et al. for IPD meta-analysis, which separates within- from between-trial estimation of treatment effects, as a way to minimize the risk of ecological fallacy (Table 3) [18].

**Table 3:** Description of each of the models used in the analysis and their differences to the gold-standard model 1

Model	Description	Difference from model 1
1	− Model for analysis of individual patient data − Estimation of the between-trial variance of the interaction between treatment effect and age − Estimation of the between-trial variance of the treatment effect − Separation of within- from between-trial interaction effects	–
2	− Model for analysis of individual patient data − Estimation of the between-trial variance of the interaction between treatment effect and age − Separation of within- from between-trial interaction effects	− No estimation of the between-trial variance of the treatment effect
3	− Model for analysis of individual patient data − Estimation of the between-trial variance of the treatment effect − Separation of within- from between-trial interaction effects	− No estimation of the between-trial variance of the interaction between treatment effect and age
4	− Model for analysis of individual patient data − Estimation of the between-trial variance of the interaction between treatment effect and age − Estimation of the between-trial variance of the treatment effect − No separation of within- from between-trial interaction effects	− No separation of within- from between-trial interaction effects
5	− Model for analysis of individual patient data − Estimation of the between-trial variance of the interaction between treatment effect and age − No separation of within- from between-trial interaction effects	− No estimation of the between-trial variance of the treatment effect − No separation of within- from between-trial interaction effects
6	− Model for analysis of individual patient data − Estimation of the between-trial variance of the treatment effect − No separation of within- from between-trial interaction effects	− No estimation of the between-trial variance of the interaction between treatment effect and age − No separation of within- from between-trial interaction effects
7	− Model for analysis of aggregate data − Estimation of the between-trial variance of the treatment effect	− Model for analysis of aggregate data − No estimation of the between-trial variance of the interaction between treatment effect and age − No separation of within- from between-trial interaction effects

*Model 1: Estimation of the between-trial variance of the interaction between treatment effect and age, estimation of the between-trial variance of the treatment effect, and separation of within- from between-trial interaction effects, using individual patient data.* Separation of within- from between-trial interaction effects was implemented as previously suggested by Riley et al. [18]. Although we are not aware it has ever been fitted in practice, this model is considered the gold-standard in this investigation because it estimates the random-effects of both interaction effect and treatment effect that exist in the simulated dataset, and also separates the within and between-trial interaction effects.

In this model, *ageC*_*ij*_ is the covariate *age*_*ij*_ centered by its mean value *m*_*i*_, in each trial, and it includes an interaction term between the treatment effect and *ageC*_*ij*_, as well as an interaction term between the treatment effect and *m*_*i*_, to separate the within- from the between-trial effects:

4.2 $$ {y}_{ij}=\alpha +\left({treat}_{ij}\ast beta. treat\right)+\left({ageC}_{ij}\ast beta. ageC\right)+\left({treat}_{ij}\ast {ageC}_{ij}\ast beta. interC\right)+\left({ageM}_i\ast beta. ageM\right)+\left({treat}_{ij}\ast {ageM}_i\ast beta. interM\right)+{u}_i+v{1}_i\ast {treat}_{ij}\ast {ageC}_{ij}+v{2}_i\ast {treat}_{ij} $$

where *y*_*ij*_ is the predicted knee OA pain measured on a 0 to 10 VAS on the *j*^th^ patient in the *i*^th^ trial, *α* is the average knee OA pain in the placebo group for patients that have an average age, *treat*_*ij*_ and *ageC*_*ij*_ are, respectively, the treatment received and age centered around the trial’s mean age of the *j*^th^ patient in the *i*^th^ trial, *ageM*_*i*_ is the mean age of patients in the *i*^th^ trial, *beta.treat* is the fixed between-group difference in knee OA pain (i.e. treatment effect), *beta.ageC* is the fixed coefficient of the centered age covariate, *beta.interC*_*i*_ is the interaction between the treatment effect and centered age in trial *i*, *beta.ageM* is the fixed coefficient of the mean value of the age covariate, *beta.interM* is the fixed interaction between the treatment effect and the mean value of age, *u*_*i*_ are the random trial effects of knee OA pain in the placebo group for patients that have an average age, *v*1_*i*_ are the random trial effects in the interaction between the treatment effect and centered age, and *v*2_*i*_ are the random trial effects in the treatment effect. This model assumes an exchangeable covariance structure between the random-effects of intercept and interaction. As previously stated, this model allows the separation of the pooled within-trial treatment-covariate interaction *beta.interC* from the between-trials interaction *beta.interM* [18]. This model has been shown to completely separate within- and between-trial effects, thus resulting in independent *beta.interC* and *beta.interM*, even when the number of subjects per study is small [18, 19]. The between-trials interaction *beta.interM* is actually the same as the output of a meta-regression, which only uses aggregate data to estimate associations between individual-level characteristics and the treatment effect (model 7 is a meta-regression) [20].

*.Model 2: Estimation of the between-trial variance of the interaction between treatment effect and age, and separation of within- from between-trial interaction effects, using individual patient data.*

4.3 $$ {y}_{ij}=\alpha +\left({treat}_{ij}\ast beta. treat\right)+\left({ageC}_{ij}\ast beta. ageC\right)+\left({treat}_{ij}\ast {ageC}_{ij}\ast beta. interC\right)+\left({ageM}_i\ast beta. ageM\right)+\left({treat}_{ij}\ast {ageM}_i\ast beta. interM\right)+{u}_i+{v}_i\ast {treat}_{ij}\ast {ageC}_{ij} $$

The parameters in model 2 are as defined previously in model 1, except that now the only random trial effects are in the interaction between the treatment effect and centered age *v*_*i*_. Again, separation of within- from between-trial interaction effects was implemented as previously suggested by Riley et al. [18].

*Model 3: Estimation of the between-trial variance of the treatment effect, and separation of within- from between-trial interaction effects, using individual patient data.*

4.4 $$ {y}_{ij}=\alpha +\left({treat}_{ij}\ast beta. treat\right)+\left({ageC}_{ij}\ast beta. ageC\right)+\left({treat}_{ij}\ast {ageC}_{ij}\ast beta. interC\right)+\left({ageM}_i\ast beta. ageM\right)+\left({treat}_{ij}\ast {ageM}_i\ast beta. interM\right)+{u}_i+{v}_i\ast {treat}_{ij} $$

The parameters in model 3 are as defined previously in model 1, except that now the only random trial effects are in the treatment effect *v*_*i*_. Again, separation of within- from between-trial interaction effects was implemented as previously suggested by Riley et al. [18].

*Model 4: Estimation of the between-trial variance of the interaction between treatment effect and age, estimation of the between-trial variance of the treatment effect, without separation of within- from between-trial interaction effects, using individual patient data*

4.5 $$ {y}_{ij}=\alpha +\left({treat}_{ij}\ast beta. treat\right)+\left({ageC}_{ij}\ast beta. ageC\right)+\left( trea{\mathrm{t}}_{ij}\ast {ageC}_{ij}\ast beta. interC\right)+{u}_i+v{1}_i\ast {treat}_{ij}\ast {ageC}_{ij}+v{2}_i\ast {treat}_{ij} $$

The parameters in model 4 are as defined previously in model 1, except that mean age is no longer part of the model. This means that the association between mean age and the outcome of knee pain, and the interaction effect between mean age and treatment allocation on the outcome of knee pain, are no longer estimated. Because mean age is not included in model 4, it does not separate the within- from between-trial interaction effects.

*Model 5: Estimation of the between-trial variance of the interaction between treatment effect and age, without separation of within- from between-trial interaction effects, using individual patient data.*

4.6 $$ {y}_{ij}=\alpha +\left({treat}_{ij}\ast beta. treat\right)+\left({ageC}_{ij}\ast beta. ageC\right)+\left({treat}_{ij}\ast {ageC}_{ij}\ast beta. interC\right)+{u}_i+{v}_i\ast {treat}_{ij}\ast {ageC}_{ij} $$

The parameters in model 5 are as defined previously in model 2, except for the parameters related to the effect of mean age within trials. Because these parameters were removed, model 5 does not separate the within- from between-trial interaction effects.

*Model 6: Estimation of the between-trial variance of the treatment effect, without separation of within- from between-trial interaction effects, using individual patient data*

4.7 $$ {y}_{ij}=\alpha +\left({treat}_{ij}\ast beta. treat\right)+\left({ageC}_{ij}\ast beta. ageC\right)+\left({treat}_{ij}\ast {ageC}_{ij}\ast beta. interC\right)+{u}_i+{v}_i\ast {treat}_{ij} $$

The parameters in model 6 are as defined previously in model 2, except for the parameters related to the effect of mean age within trials. Because these parameters were removed, model 6 does not separate the within- from between-trial interaction effects.

*Model 7: Estimation of the between-trial variance of the treatment effect using aggregate data, which can only estimate the between-trial interaction effects*

4.8 $$ {\displaystyle \begin{array}{c}{y}_i=\alpha +\left({ageM}_i\ast beta. age\right)+{u}_i\\ {}{u}_i\sim N\left(0,{\sigma}^2\right)\end{array}} $$

where *y*_*i*_ is the predicted between-group difference in knee OA in the *i*^th^ trial, *α* is the fixed between-group difference in knee OA pain, *ageM*_*i*_ is the mean age of patients in the *i*^th^ trial, beta.age is the fixed interaction effect between the treatment effect and the mean age of patients in the *i*^th^ trial, and *u*_*i*_ is the random effect of the between-group difference in knee OA pain.

### Performance measures

Bias, coverage, empirical and model-based standard errors, and the relative error in the model standard error for the estimand *θ* were assessed. Estimation of performance measures and their Monte Carlo Standard Error (MCSE) was conducted as described by Morris et al. [15]. Bias was considered acceptable if it was ≤10 times lower than the simulated interaction effect of − 0.01. Coverage of the 95% confidence interval of the estimated interaction effect was assessed. The explanation on how each of the performance measures were calculated are presented in Additional file 1: Appendix B.

#### Sample size calculation

The simulation was powered to have a precise estimate of coverage. With an expected coverage of 95%, and a desired MCSE of coverage of 0.5%, 1900 repetitions would be needed [15]. It was then decided to use 2000 repetitions for each simulation, which results in a small MCSE for the estimate of all performance measures (Table 4).

**Table 4:** Largest Monte Carlo Standard Errors (MCSE) expected with 2000 simulations for each of the performance measures of interest

Estimate	MCSE
Bias	≤0.03
Coverage	≤0.05
Empirical SE	≤0.0005
Model SE	≤0.0005
Relative error in the model SE	≤0.018

*SE* standard error

#### Software used to analyse the performance of the simulated data

All analyses were conducted in Stata 14.2.

## Results

In this section, we focus on results pertaining to the large but plausible between-trial variation of 0.05 in the interaction effect since our main purpose is to assess model performance when this variation is taken into account. Results of other scenarios using a between trial variation of different magnitude are presented in the Additional file 1.

### Results of simulations assuming a large between-trial variation of 0.05 in the interaction effect

**Fig. 1:**
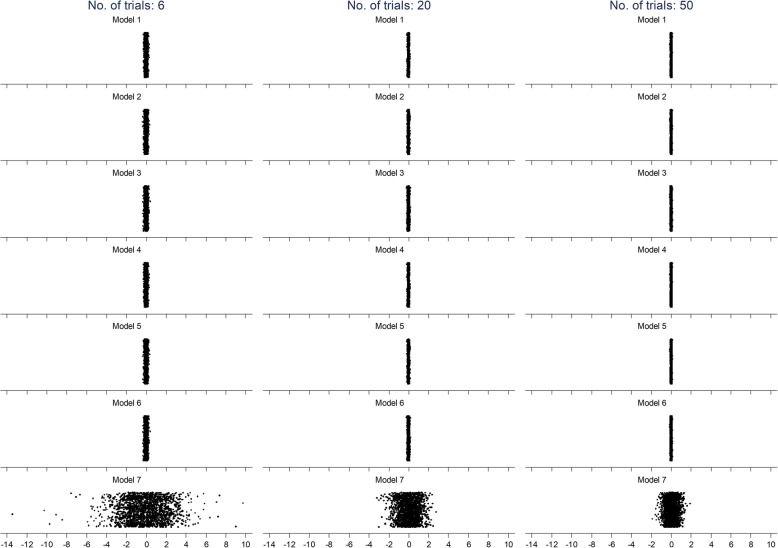
Scatter of interaction effects from 2000 repetitions for each of the models for analyses with 6, 20, and 50 trials

Figure 1 shows the interaction effects estimated in each of the 2000 repetitions according to all models for analyses with 6, 20, and 50 trials and the large between-trial variation of 0.05 in the interaction effect. It can be seen from this graph that the interaction effects from models that used IPD have a much narrower scatter than those from model 7, the only model that used AD. Through visual inspection, the mean value of the interaction effects appears similar across all models. Also, the scatter of interaction effects decreases as the number of trial increases, for all of the models.

**Fig. 2:**
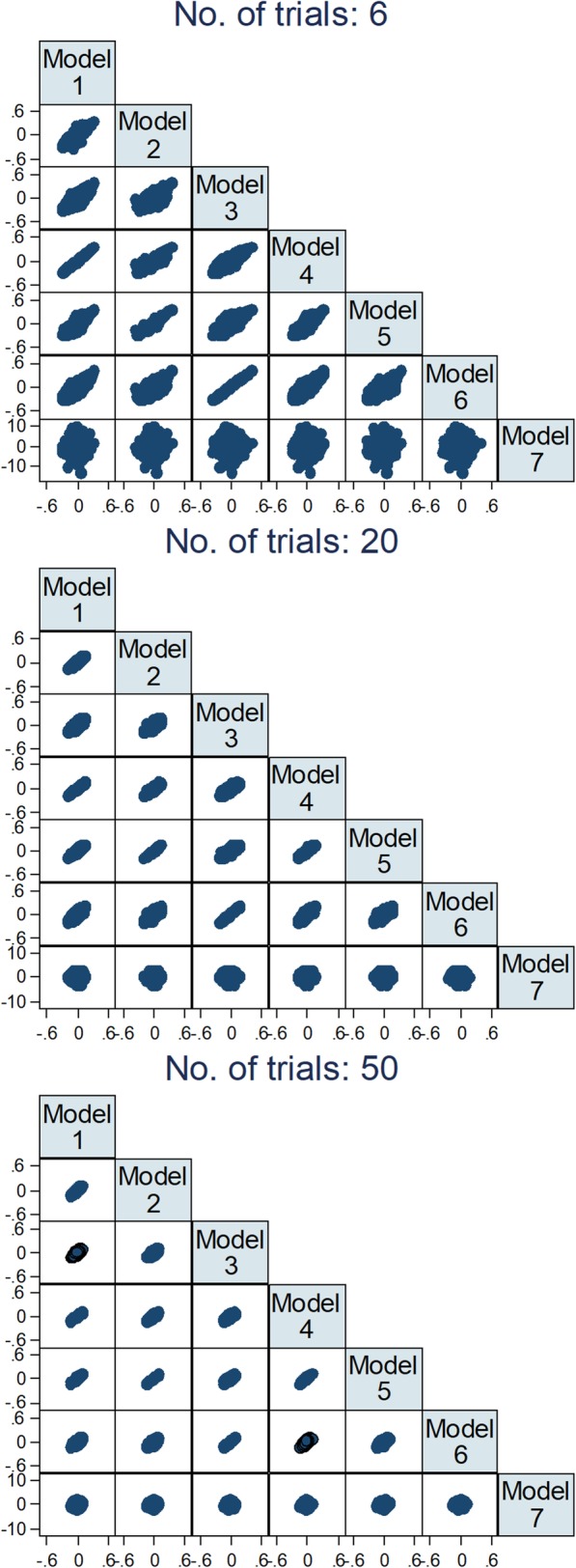
Comparison between models of estimates of interaction effects across 2000 repetitions for 6, 20, and 50 trials

Figure 2 shows scatter plots of interaction effects of all models plotted against each other. It can be seen that there is a considerable agreement between all models that used IPD, with higher agreement between models that made the same assumptions regarding between-trial variation of effects. It can also be seen that there is very low correlation between results from models using IPD and the model 7, which used AD. Although increasing the number of trials decreased the variation of estimates for all models, it did not have an important influence in the correlation between the results of different models.

**Fig. 3:**
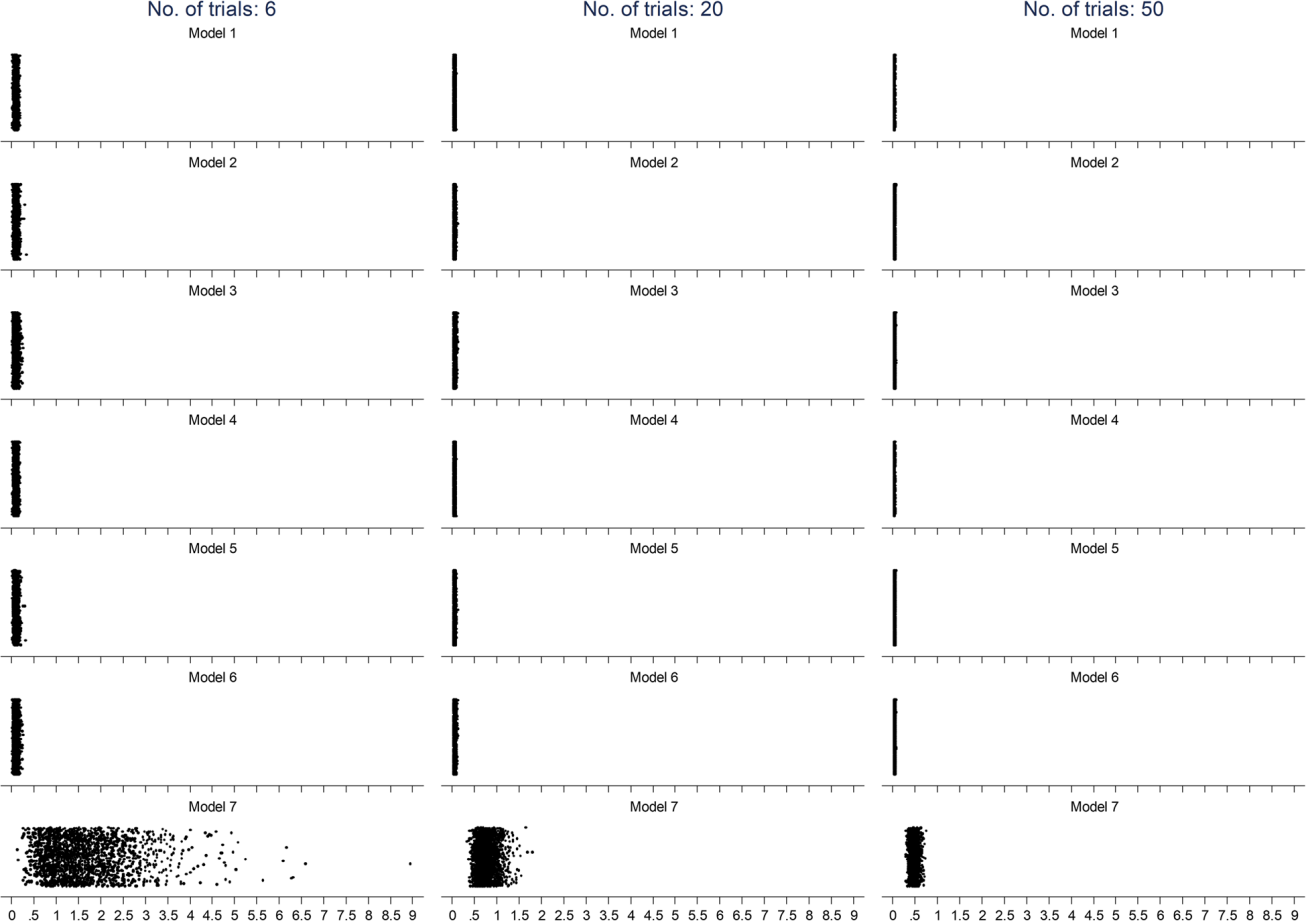
Scatter of standard error of the interaction effects for each of the models for analyses with 6, 20, and 50 trials

Figure 3 shows the standard error of the interaction effects according to all models for analyses with 6, 20, and 50 trials. As with the interaction effects, this graph shows that the scatter of standard errors is narrower in models that used IPD than model 7, which used AD. Again, as with the interaction effect, the scatter of the standard error decreases as the number of trials increases. However, different than what was observed with the interaction effects, the mean value of the standard error in model 7 was systematically larger than those in models that used IPD.

**Fig. 4:**
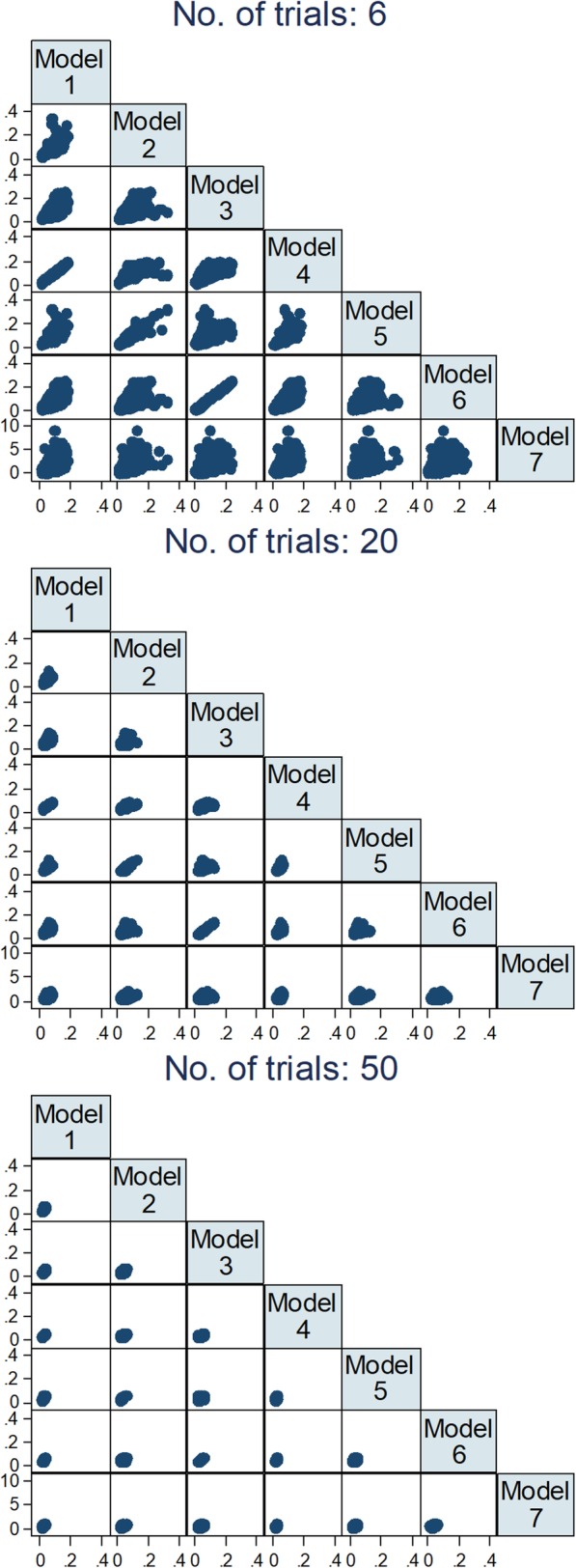
Comparison between models of estimates of standard errors of interaction effects across 2000 repetitions for 6, 20, and 50 trials

Figure 4 shows scatter plots of the standard error of interaction effects of all models plotted against each other. As previously observed for interaction effects, there is a higher correlation in standard errors between models that used the same assumptions regarding the estimation of between-trial variance. Although there was a clear correlation in standard errors between models that used IPD, this correlation is lower than the one observed for interaction effects. As with interaction effects, an increase in the number of trials did not increase the correlation of standard errors between models, and the correlation between models using IPD and model 7 was very low.

Table 5 shows the estimated bias in the interaction effect in each of the models for all investigated number of trials. Bias was most influenced by whether the between-trial variation was estimated for the interaction effect, the treatment effect, or both. A separate estimation of within- and between-trial effects did not play an important role. It can be seen that bias tends to decrease as the number of trials increases for all of the models, with a more extensive difference in model 7, which used aggregate data. While all other models had a satisfactory bias with a lower number of trials, model 7 had a bias effect of − 0.065, which is 6.5 times the simulated interaction effect of − 0.01. Model 7 only has an acceptable bias of 0.0007 when 50 trials are included in the analysis. Among the models that used IPD (i.e. models 1–6), only models that modelled exclusively the between-trial variation of the interaction effect (i.e. models 2 and 5) performed well when the number of trials was only 6. Model 2, which modelled the between-trial variation in the interaction effect and separated within- and between-trial effects, had the lowest bias (− 0.00006). Model 5, which modelled the between-trial variation in interaction effects but did not separate within- from between-trial effects, had also an acceptable bias of 0.00038. With 10 trials included in the analysis, the magnitude of bias becomes acceptable for models that exclusively modelled the between-trial variation of the treatment effect (i.e. models 3 and 6). Only when 16 trials were included in the analysis that models 1 and 4, which modelled between-trial variation for both treatment effects and interaction effects, achieved acceptable levels of bias. Finally, the MCSE was relatively large for all bias estimates.

**Table 5:** Bias in the interaction effect between treatment effect and covariate (age) in each of the models in the presence of high but plausible heterogeneity in the interaction effect

No. of trials	Model 1	Model 2	Model 3	Model 4	Model 5	Model 6	Model 7
6	0.00179 (0.09149)	−0.00006 (0.09595)	0.00323 (0.10316)	0.00179 (0.09149)	0.00038 (0.09622)	0.00323 (0.10316)	−0.06454 (1.87747)
10	−0.00101 (0.07082)	−0.00079 (0.07411)	− 0.00070 (0.08183)	− 0.00101 (0.07082)	− 0.00072 (0.07410)	−0.00070 (0.08183)	0.01286 (1.22375)
16	−0.00013 (0.05543)	−0.00060 (0.05795)	− 0.00094 (0.06589)	−0.00014 (0.05543)	− 0.00050 (0.05800)	−0.00094 (0.06589)	0.01857 (0.93068)
20	−0.00049 (0.05012)	−0.00102 (0.05260)	− 0.00119 (0.05889)	−0.00049 (0.05012)	− 0.00097 (0.05254)	−0.00119 (0.05889)	− 0.02249 (0.81903)
26	−0.00051 (0.04289)	− 0.00079 (0.04524)	0.00012 (0.05107)	− 0.00051 (0.04289)	−0.00076 (0.04517)	0.00012 (0.05107)	−0.01126 (0.67902)
30	−0.00013 (0.04099)	0.00051 (0.04331)	0.00018 (0.04889)	−0.00013 (0.04099)	0.00049 (0.04323)	0.00018 (0.04889)	0.00940 (0.62751)
40	0.00034 (0.03480)	0.00073 (0.03687)	0.00050 (0.04180)	0.00034 (0.03480)	0.00074 (0.03686)	0.00050 (0.04180)	0.01953 (0.55119)
50	−0.00010 (0.03198)	−0.00005 (0.03364)	− 0.00013 (0.03806)	−0.00010 (0.03198)	− 0.00004 (0.03368)	−0.00013 (0.03806)	0.00074 (0.48328)

Values in brackets are Monte Carlo standard errors

Table 6 shows the observed coverage of the interaction effect between treatment effect and age in each of the models. With only 6 trials, coverage was best with model 7, with an observed coverage of 94.4%, while all models using IPD had an observed coverage below 90%. Coverage was worst with models that exclusively estimated the between-trial variance in treatment effects, with observed values around 83.4%. As the number of trials increased, coverage also increased, but models using IPD consistently overestimated coverage, with observed coverage values always lower than the nominal value. For models using IPD, those that estimated the between-trial variation in the interaction effect had acceptable coverage levels when at least 20 trials were included in the analysis. Models 3 and 5 only had an acceptable coverage when 40 trials were included in the analysis. Among models using IPD, those that estimated the between-trial variation in the interaction effect had consistently better coverage than models that did not. Coverage was consistently accurate for model 7 across all numbers of trials. Finally, all coverage estimates were precise, with low MCSEs.

**Table 6:** Observed coverage of the interaction effect between treatment effect and covariate (age) in each of the models, with a nominal coverage of 95% in the presence of high but plausible heterogeneity in the interaction effect

No. of trials	Model 1	Model 2	Model 3	Model 4	Model 5	Model 6	Model 7
6	0.89400 (0.00688)	0.88500 (0.00713)	0.83850 (0.00823)	0.89450 (0.00687)	0.88550 (0.00712)	0.83850 (0.00823)	0.94350 (0.00516)
10	0.92250 (0.00598)	0.92100 (0.00603)	0.88950 (0.00701)	0.92250 (0.00598)	0.92150 (0.00601)	0.88950 (0.00701)	0.95250 (0.00476)
16	0.92850 (0.00576)	0.92600 (0.00585)	0.90800 (0.00646)	0.92900 (0.00574)	0.92700 (0.00582)	0.90800 (0.00646)	0.94900 (0.00492)
20	0.93350 (0.00557)	0.93900 (0.00535)	0.92350 (0.00594)	0.93400 (0.00555)	0.93450 (0.00553)	0.92350 (0.00594)	0.94050 (0.00529)
26	0.94150 (0.00525)	0.94300 (0.00518)	0.93250 (0.00561)	0.94150 (0.00525)	0.94250 (0.00521)	0.93250 (0.00561)	0.95300 (0.00473)
30	0.93150 (0.00565)	0.93300 (0.00559)	0.91750 (0.00615)	0.93150 (0.00565)	0.93400 (0.00555)	0.91750 (0.00615)	0.94900 (0.00492)
40	0.94650 (0.00503)	0.93800 (0.00539)	0.93250 (0.00561)	0.94650 (0.00503)	0.93700 (0.00543)	0.93250 (0.00561)	0.94450 (0.00512)
50	0.94700 (0.00501)	0.94950 (0.00490)	0.93400 (0.00555)	0.94700 (0.00501)	0.94900 (0.00492)	0.93400 (0.00555)	0.95400 (0.00468)

Values in brackets are Monte Carlo standard errors

**Fig. 5:**
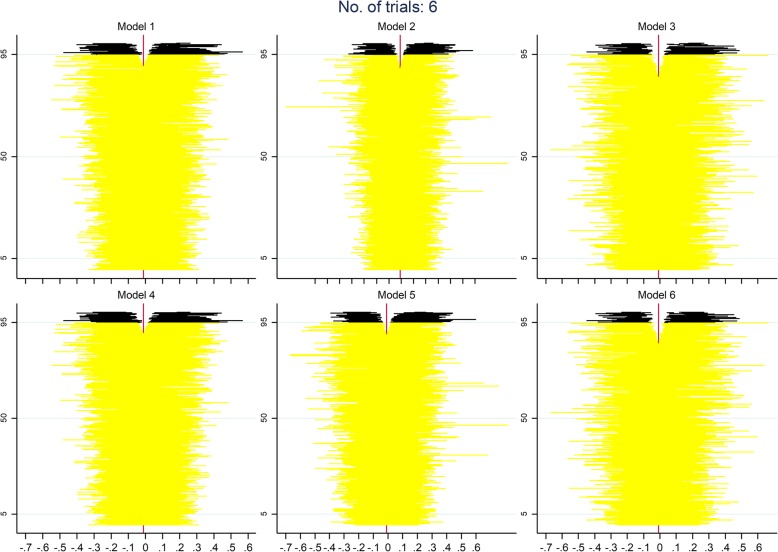
Adapted zip plots of the 2000 confidence intervals for all IPD models for analyses with 6 trials

**Fig. 6:**
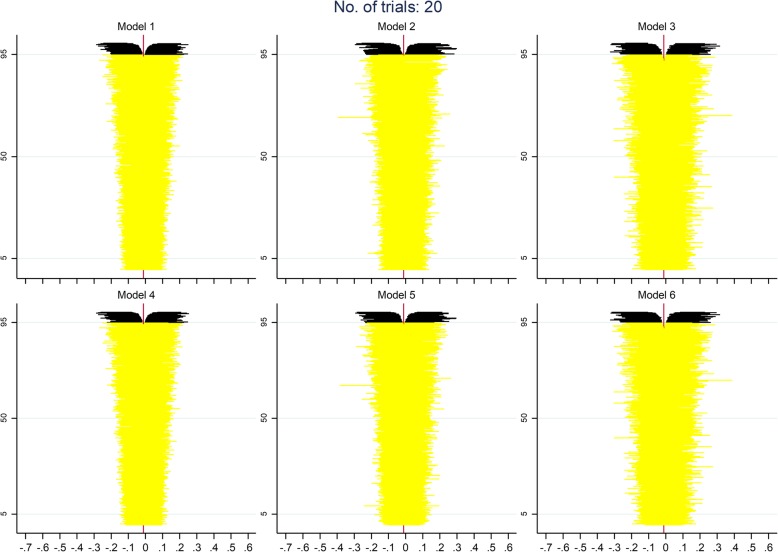
Adapted zip plots of the 2000 confidence intervals for all IPD models for analyses with 20 trials

**Fig. 7:**
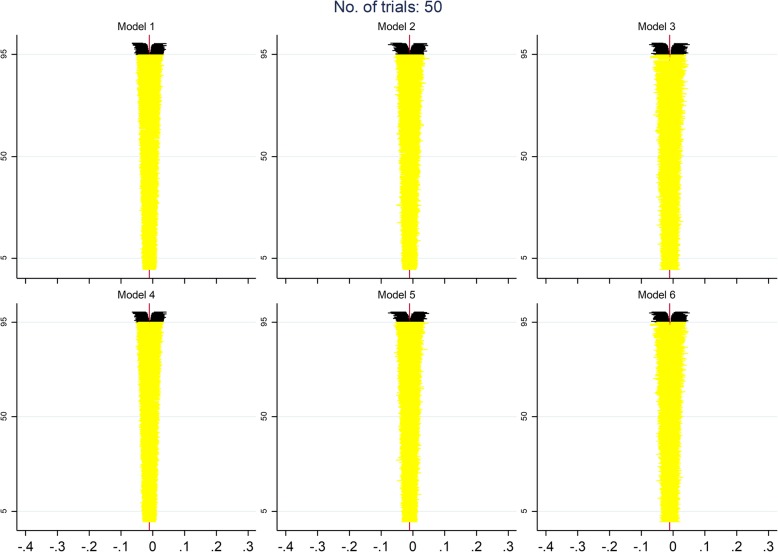
Adapted zip plots of the 2000 confidence intervals for all IPD models for analyses with 50 trials

Figures 5, 6 and 7 display zip plots of the 2000 confidence intervals for all IPD models for analyses with 6, 20, or 50 trials (see Additional file 1: Appendix B for an explanation on how to interpret zip plots). We can readily see that all models using IPD had insufficient coverage when only 6 trials were included in the analysis, with models 3 and 6, which only addressed the between-trial variance of the treatment effect, having the worst coverage. With 20 trials included in the analysis, we can see that coverage is appropriate for most models, but that models 3 and 6 are still slightly overestimating the nominal coverage. With 50 trials included in the analysis it becomes clear that all models using IPD have an appropriate coverage.

Table 7 shows how each model performed in the estimation of standard errors, for different numbers of trials included in the analysis. In general, both empirical and model SEs were smaller in models 1 and 4, where the between-trial variance of treatment effects and interaction effects are estimated and allowed for. Models 2 and 5, which estimate and allow for the between-trial variance in interaction effects only, had the second smallest empirical and model SEs. Among models using IPD, models 3 and 6, which estimated and allowed for the between-trial variance in treatment effects, but ignored the between-trial variance in interaction effects, yielded the largest SEs, and thus have the lowest precision. Model 7, the only model that used AD, yielded clearly higher SEs than the other models. The difference in SEs between models using IPD were small. These findings were not affected by the number of trials included in the analysis, or whether within- and between-trial effects were modelled separately or together. When assessing the error in the model SE in relation to the empirical SE, the model SE was most accurate if the model also estimated and accounted for the between-trial variance in interaction effects (models 1,2,4,5), while models that ignored this had a consistently higher relative error in the model SE (models 3,6,7). This pattern was consistent regardless of the number of trials included in the analysis, or if within- and between-trial effects were modelled separately or together.

**Table 7:** Standard error of the interaction effect between treatment effect and covariate (age) in each of the models in the presence of high but plausible heterogeneity in the interaction effect

No. of trials	Performance measure	Model 1	Model 2	Model 3	Model 4	Model 5	Model 6	Model 7
6	Empirical SE	0.09147 (0.00145)	0.09595 (0.00152)	0.10311 (0.00163)	0.09148 (0.00145)	0.09621 (0.00152)	0.10311 (0.00163)	1.87636 (0.02968)
	Model SE	0.09125 (0.01059)	0.09486 (0.01456)	0.09568 (0.01640)	0.09125 (0.01059)	0.09528 (0.01454)	0.09568 (0.01640)	1.81917 (177.01665)
	Relative error in model SE	−0.24168 (1.72411)	−1.13917 (1.77644)	−7.20306 (1.70537)	− 0.24417 (1.72406)	− 0.97397 (1.77358)	−7.20303 (1.70537)	−3.04755 (2.09350)
10	Empirical SE	0.07081 (0.00112)	0.07410 (0.00117)	0.08183 (0.00129)	0.07081 (0.00112)	0.07410 (0.00117)	0.08183 (0.00129)	1.22368 (0.01935)
	Model SE	0.07072 (0.00378)	0.07411 (0.00540)	0.07776 (0.00676)	0.07072 (0.00378)	0.07412 (0.00534)	0.07776 (0.00676)	1.23611 (36.09575)
	Relative error in model SE	−0.13873 (1.66686)	0.00346 (1.71505)	−4.96244 (1.65106)	−0.13683 (1.66689)	0.03390 (1.71257)	−4.96244 (1.65106)	1.01558 (1.86656)
16	Empirical SE	0.05543 (0.00088)	0.05794 (0.00092)	0.06588 (0.00104)	0.05543 (0.00088)	0.05800 (0.00092)	0.06588 (0.00104)	0.93050 (0.01472)
	Model SE	0.05606 (0.00144)	0.05883 (0.00200)	0.06377 (0.00321)	0.05606 (0.00144)	0.05867 (0.00196)	0.06377 (0.00321)	0.92118 (10.53523)
	Relative error in model SE	1.13187 (1.65210)	1.53029 (1.68156)	−3.21023 (1.64405)	1.13079 (1.65209)	1.15699 (1.67359)	−3.21024 (1.64405)	−1.00201 (1.70189)
20	Empirical SE	0.05012 (0.00079)	0.05259 (0.00083)	0.05887 (0.00093)	0.05012 (0.00079)	0.05253 (0.00083)	0.05887 (0.00093)	0.81872 (0.01295)
	Model SE	0.05005 (0.00089)	0.05253 (0.00128)	0.05719 (0.00207)	0.05005 (0.00089)	0.05253 (0.00130)	0.05719 (0.00207)	0.80839 (6.13561)
	Relative error in model SE	−0.14153 (1.61861)	−0.10115 (1.64049)	−2.86836 (1.62743)	−0.14211 (1.61860)	0.00514 (1.64349)	−2.86838 (1.62743)	−1.26192 (1.66352)
26	Empirical SE	0.04289 (0.00068)	0.04523 (0.00072)	0.05107 (0.00081)	0.04289 (0.00068)	0.04517 (0.00071)	0.05107 (0.00081)	0.67892 (0.01074)
	Model SE	0.04404 (0.00054)	0.04615 (0.00074)	0.05064 (0.00127)	0.04404 (0.00054)	0.04612 (0.00074)	0.05064 (0.00127)	0.69302 (3.18236)
	Relative error in model SE	2.69132 (1.65593)	2.02827 (1.65925)	−0.83126 (1.64152)	2.69167 (1.65594)	2.10172 (1.65950)	−0.83126 (1.64152)	2.07716 (1.68652)
30	Empirical SE	0.04099 (0.00065)	0.04331 (0.00068)	0.04889 (0.00077)	0.04099 (0.00065)	0.04323 (0.00068)	0.04889 (0.00077)	0.62743 (0.00992)
	Model SE	0.04073 (0.00040)	0.04282 (0.00058)	0.04681 (0.00092)	0.04073 (0.00040)	0.04278 (0.00058)	0.04681 (0.00092)	0.63396 (2.30260)
	Relative error in model SE	−0.63932 (1.59872)	−1.13154 (1.60574)	−4.27073 (1.57354)	−0.64006 (1.59871)	−1.03999 (1.60736)	−4.27072 (1.57354)	1.03939 (1.66192)
40	Empirical SE	0.03480 (0.00055)	0.03687 (0.00058)	0.04180 (0.00066)	0.03480 (0.00055)	0.03685 (0.00058)	0.04180 (0.00066)	0.55085 (0.00871)
	Model SE	0.03545 (0.00022)	0.03722 (0.00034)	0.04099 (0.00053)	0.03545 (0.00022)	0.03719 (0.00034)	0.04099 (0.00053)	0.54445 (1.23398)
	Relative error in model SE	1.87182 (1.63128)	0.94769 (1.63044)	−1.92503 (1.59642)	1.87298 (1.63130)	0.93484 (1.62997)	−1.92503 (1.59642)	−1.16099 (1.60819)
50	Empirical SE	0.03198 (0.00051)	0.03364 (0.00053)	0.03806 (0.00060)	0.03198 (0.00051)	0.03368 (0.00053)	0.03806 (0.00060)	0.48328 (0.00764)
	Model SE	0.03165 (0.00014)	0.03338 (0.00021)	0.03673 (0.00034)	0.03165 (0.00014)	0.03337 (0.00021)	0.03673 (0.00034)	0.48219 (0.75386)
	Relative error in model SE	−1.03205 (1.58095)	−0.79436 (1.59442)	−3.48739 (1.56226)	−1.03213 (1.58094)	−0.91692 (1.59272)	−3.48739 (1.56226)	− 0.22640 (1.61322)

Values in brackets are Monte Carlo standard errors

Table 8 shows the mean squared error of each model for different numbers of trials included in the analysis. It can be seen that the mean squared error decreased as the number of trials included in the analysis increased, that models using IPD have lower mean squared errors than the model that used AD, and that models that accounted for the between-trial variance in the interaction effect had a lower mean squared error than those that did not. The most important factor leading to higher values of the mean squared error was the use of AD for the analysis. The model that used AD had considerably larger mean squared errors than models that used IPD. Although the mean squared error decreased as the as the number of trials included in the analysis increased, it was still considerably higher for the model using AD even when 50 trials were included in the analysis. Among models that used IPD, differences in mean squared error seem negligible, even though models that accounted for the between-trial variance in the interaction effect had lower mean squared errors than those that did not.

**Table 8:** Mean squared error of the interaction effect between treatment effect and covariate (age) in each of the models in the presence of high but plausible heterogeneity in the interaction effect

No. of trials	Model 1	Model 2	Model 3	Model 4	Model 5	Model 6	Model 7
6	0.00837 (0.00027)	0.00920 (0.00029)	0.01064 (0.00034)	0.00837 (0.00027)	0.00925 (0.00029)	0.01064 (0.00034)	3.52311 (0.18382)
10	0.00501 (0.00016)	0.00549 (0.00017)	0.00669 (0.00022)	0.00501 (0.00016)	0.00549 (0.00017)	0.00669 (0.00022)	1.49681 (0.05581)
16	0.00307 (0.00010)	0.00336 (0.00011)	0.00434 (0.00014)	0.00307 (0.00010)	0.00336 (0.00011)	0.00434 (0.00014)	0.86574 (0.03416)
20	0.00251 (0.00008)	0.00277 (0.00009)	0.00347 (0.00011)	0.00251 (0.00008)	0.00276 (0.00009)	0.00347 (0.00011)	0.67048 (0.02303)
26	0.00184 (0.00006)	0.00205 (0.00007)	0.00261 (0.00008)	0.00184 (0.00006)	0.00204 (0.00007)	0.00261 (0.00008)	0.46083 (0.01572)
30	0.00168 (0.00005)	0.00187 (0.00006)	0.00239 (0.00008)	0.00168 (0.00005)	0.00187 (0.00006)	0.00239 (0.00008)	0.39357 (0.01326)
40	0.00121 (0.00004)	0.00136 (0.00004)	0.00175 (0.00005)	0.00121 (0.00004)	0.00136 (0.00004)	0.00175 (0.00005)	0.30366 (0.00986)
50	0.00102 (0.00003)	0.00113 (0.00004)	0.00145 (0.00004)	0.00102 (0.00003)	0.00113 (0.00004)	0.00145 (0.00004)	0.23345 (0.00767)

Values in brackets are Monte Carlo standard errors

### Results of simulations assuming a null, small, or implausible large between-trial variation in the interaction effect

Results of the simulation with a between-trial variance of zero, 0.005, or 0.5 are shown in Additional file 1: Appendices C, D and E. Findings of our simulation with a null or small between-trial variance were similar to the simulations with a large but plausible between-trial variance, but with much smaller differences between models using IPD. Differences in performance between models using IPD and the model using AD decreased as the between-trial variation decreased. The difference in all performance measures between the models using IPD were consistently small, with a pattern indicating that models that allowed for the between-trial variance in the interaction effect had lower bias, and better precision and coverage. Again, similar to the simulations assuming a large but plausible between-trial variance, the model using AD had a generally worse performance than models using IPD. Although this difference in performance decreased as the number of trials increased, the model using AD still had a worse performance than models using IPD even when 50 trials were included in the analysis, albeit unimportant differences. No relevant difference was observed between models that separated within- and between-trial associations and models that did not separate them.

Findings of our simulation with an implausible large between-trial variance were similar to those from the simulations with a large but plausible between-trial variance. However, the difference in performance between models was accentuated with the implausible large between-trial variance, mainly when the number of trials included in the analysis was low.

### Results of simulations assuming a null interaction effect

Results from analyses assuming a null interaction effect were remarkably similar to those from analyses assuming an interaction effect of − 0.01 (results available on request).

## Discussion

### Main findings

In this simulation study, the performance of seven different meta-regression models for the pooling across trials of interaction effects between a patient-level characteristic and the treatment effect were compared and it was observed that a better performance was obtained in models that used IPD and that allowed for between-trial variation of the interaction effect. Although models that allowed for between-trial variation of the interaction effect had a better performance than models that did not allow for this variation, other model or dataset characteristics played a more important role in model performance. The main factor influencing the performance of models was whether they used IPD or AD. Models based on IPD generally had acceptable levels of bias that were ten times smaller than the simulated interaction effect of − 0.01 when ten or more trials were included in the analysis. Biases in the model using AD were approximately two to six times larger than the simulated interaction effect, and was only acceptable when 50 trials were included in the analysis. The model that used AD also had a considerably lower precision than all models that used IPD, especially when a low number of trials was included in the analysis, although coverage of AD models was generally appropriate even with a low number of trials in the analysis. The second most important factor influencing the performance of the models was the number of trials included in the analysis. In general, as the number of trials included in the analysis increased, the performance of all models improved. Another important factor that influenced model performance was the magnitude of heterogeneity. Differences in performance between models was more evident in the presence of high heterogeneity (i.e. large between-trial variation of the interaction effect), which is not surprising given only certain models acknowledge the possibility of heterogeneity in interaction effects. The performance of models was also influenced, to a lesser degree, by whether the model allowed for between-trial variation of the interaction effect. Models that used IPD and allowed for between-trial variation of the interaction effect had the best performance, generally with lower bias, better coverage, and higher precision than all other models. The model with the worst performance among IPD models was the one that allowed for between-trial variation in treatment effects but ignored the between-trial variation in interaction effects, and it is likely the most commonly used model in practice for IPD analyses. The gold-standard model, which was a model that allowed for between-trial variation in both treatment and interaction effects and used IPD, did not perform well when only ten trials were analysed. No relevant difference was observed between models that separated within- and between-trial associations and models that did not separate them, which is unsurprising, as differences in within- and between-trial associations was not simulated in the dataset (i.e. ecological biases were not simulated). Likewise, the presence of an interaction effect did not seem to influence results, since results of analyses assuming a null interaction effect or an interaction effect of − 0.01 were remarkably similar.

### Practical implications

This study shows that when investigating interaction effects in meta-regressions, using IPD with a model that accounts for the between-trial variation of interaction effects is likely to result in more accurate results, with less bias, better coverage, and higher precision, especially in the presence of high heterogeneity of interaction effects. Not enough importance has been given to modelling the between-trial variation of interaction effects when pooling them across trials, with technical papers about regression models mainly focusing on modelling the between-trial variation of the treatment effect [18, 20, 21]. The reason for this, perhaps, is because although modelling the between-trial variation in interaction effect improves the accuracy of results, this improvement seems rather irrelevant in the absence of high heterogeneity. Nevertheless, it is important that the between-trial variation in interaction effects is reported, perhaps together with prediction intervals around the interaction effect, so that readers can better appreciate how high or how low this variation is [13]. In practice, modelling both between-trial variations, of the interaction effect and treatment effect, may lead to model convergence problems, especially if a low number of trials is included in the analysis. The lowest number of trials used in the present simulation analysis was six, and no convergence problems were observed. Whenever convergence problems are observed, researchers may try modelling only the between-trial variation of interaction effects, and only if the problem persists, try to model only the between-trial variation of treatment effects. Researchers should also always give precedence to IPD over AD when conducting regression analyses to pool interaction effects across trials, especially if the number of trials is below 50, which is often the case. Our simulation analysis indicates that if 50 trials with AD are included in a meta-regression, results may be comparable to regression analyses using IPD, in terms of bias and coverage. However, even when 50 trials with AD were included in the meta-regression, precision was still considerably lower than a regression analysis with 50 trials with IPD. Thus, since most meta-regression analyses include only a few trials, every effort should be made to access IPD when the main goal is to pool subgroup effects across trials. Interestingly, the model using AD had a better coverage than models using IPD when the number of trials analysed was low. This is likely due to the use of a Knapp-Hartung approach for estimating the variance of the mean interaction effect estimate in the AD model, which corrects the between study variance to account for its uncertainty [22]. This correction, developed for AD models, has been shown to have a better coverage performance of the 95% confidence interval than other approaches that assume that the between study variance is known [23]. Moreover, using IPD when investigating subgroup effects in a regression analysis will not only lead to results with better statistical properties, but is also necessary to avoid ecological fallacy [3]. Finally, because IPD is often only available for a small proportion of the trials included in a meta-analysis, an alternative approach would be to combine the evidence from IPD and AD when conducting such regression analyses, which is beyond the scope of the present simulation study [24].

### Strengths and limitations

The main strength of this simulation analysis is that it mimicked characteristics commonly found in meta-analyses. This simulation analysis allowed for a wide range of number of trials included in the analysis, for a varying number of patients included across trials included in the analysis with a balanced mix of small and large trials, and for scenarios with small and large between-trial variation in the interaction effect. Because the motivation to conduct this investigation is future research of osteoarthritis treatments, the simulated dataset used representative magnitude and within- and between-trial variations of treatment effects and average patient age observed in osteoarthritis trials [16]. Moreover, the use of simulated datasets allowed us to observe the statistical properties of different meta-regression models without the potential confounding influence of factors such as biases due to low methodological quality, which could hinder the proper interpretation of the results.

The main limitation of the present investigation is the definition of small and large heterogeneity. Heterogeneity is of great relevance to this investigation, since its main goal is to compare models that appropriately address it or not. The cut-offs used to define small and large but plausible heterogeneity were arbitrarily chosen to represent real datasets of osteoarthritis treatments. However, one may argue that even the definition of a small heterogeneity may not be so small, and that the large but plausible heterogeneity may be unusually large for datasets of real osteoarthritis clinical trials. This is done consciously to err on the safe side. That is, because the performance of this models can only be different in the presence of heterogeneity, cut-offs of small and large heterogeneity were chosen so that at least some heterogeneity was always present. We also analysed a simulated dataset with an implausible high heterogeneity for a better understanding of how models perform across different values of between-trial variance.

Another limitation is that results are only generalizable to trials using continuous outcomes, and arguably, only to osteoarthritis trials. However, it is expected that similar results are observed in trials with binary outcomes. In addition, although the performance of models that separate within- from between-trial associations was compared to the performance of models that do not, the dataset was not simulated to have different within- and between-trial associations of age and treatment effect. Thus, it is not surprising that there was no difference between models that separated or not these two types of associations. Likewise, although simulated datasets included small and large trials, we did not simulate small-study effects [17]. Discordant results between small and large trials may play a major role in the results of a random-effects meta-analysis [11, 25]. Finally, IPD meta-analysis of continuous outcomes are less subject to overfitting and may accommodate a larger number of covariates to account for potential confounding than IPD meta-analysis of binary outcomes or meta-regression in general [26–28]. Since the main purpose was to investigate the practical implications of adding random-effects to an interaction test in an IPD meta-analysis, we did not extended our simulations to scenarios where multiple confounding variables may be present.

### Previous research

This is the first investigation in continuous outcomes to compare the models that used IPD and AD while accounting for between-trial variation in the interaction effect. Lambert et al. conducted a simulation study to compare fixed-effect regression analyses using IPD or AD to estimate the interaction between treatment effects and patient-level characteristics [6]. They reported that AD can be used in meta-regression if a large number of large trials is available, and concluded that AD can be used to adequately estimate an interaction between the treatment effect and trial-level characteristics, but that IPD would be needed to accurately estimate an interaction between treatment effects and patient-level characteristics. Similarly to their study, the current simulation study compared IPD and AD for the estimation of an interaction between treatment effects and patient-level characteristics, but extended the investigation of Lambert et al. by comparing random-effects models. Similar to their findings based on fixed-effect models, our investigation in random-effects models concluded that AD can be used to estimate interaction effects if a large number of trials is available, but that IPD is needed when estimating an interaction between treatment effects and patient-level characteristics, mainly due to problems with ecological fallacy.

Stewart et al. conducted a similar investigation to ours using a real dataset of 24 RCTs evaluating antiplatelet agents for the prevention of pre-eclampsia in pregnancy, a binary outcome [21]. They compared three different models that were similar to the ones used in the present investigation: one model that had random-effects for the treatment effect but had a fixed-effect for the interaction effect (corresponding to model 6 in the present study), one model with the same characteristics of the previous one but that separated the within- and between-trials associations (corresponding to model 3 in the present study), and one model that had random-effects for both the treatment effect and interaction effect and did not separate within- and between-trials associations (corresponding to model 4 in the present study). The authors concluded that results across these models were virtually the same and led to the same conclusions. This is somewhat different from the conclusions of the present study, as it was noticed that albeit small, there were improvements in the accuracy of results when the model allowed for random-effects of the interaction between treatment effect and covariate. This difference in conclusions may have happened for different reasons. The first reason is that they had a fixed number of 24 trials in their analysis, and as shown in our analysis, differences in performance between models are most evident with a lower number of trials. Another reason is that there was perhaps a low between-trial variation in the interaction effect in their dataset. It was shown in this simulation that the lower the between-trial variance, the less evident is the difference in performance between models. Because we conducted a simulation study which allowed us to vary several parameters that could potentially play a role in the results of meta-analyses, as opposed to the study of Stewart et al. which had only fixed parameters, we could observe and compare the performance of all seven models throughout a wide range of values from these parameters. Finally, this simulation analysis was based on continuous outcomes, while the analysis of Stewart et al. was based on a binary outcome. It is unlikely that the difference in results observed between the studies can be explained by this, however.

### Future research

Several future research questions could develop from the current investigation. For instance, future research could be conducted to develop and investigate whether a model based on AD and that allows for between-trial variation in interaction effects has a better performance than the model based on AD that only allows for the between-trial variation in the treatment effect. Future research could also explore whether having lower or higher number of trials than what was investigated in the present study could play a role in the performance of these models. Future research could also investigate if similar results are observed with binary outcomes and if the performance of the models change in the presence of ecological fallacy, small study effects, or confounding from single or multiple other sources. Future research should also investigate more thoroughly the performance of different models for the estimation of the between trial variation in the interaction effect, including an assessment of how this estimation or lack thereof may influence the estimation of other parameters in the model (e.g. is the between-trial variation of the treatment effect overestimated if the interaction term is not included in the model?). Also, the current study only investigates a single two-way interaction, but future studies may investigate more complex interactions, such as a three-way interaction. Finally, future research should consider investigating the use of permutation tests to control for false positive rates to compare results of different regression models using these tests [27].

## Conclusions

The results of the present investigation indicate that IPD models that allow for the between-trial variation in interaction effects should be given preference over models that only allow for between-trial variation in treatment effects when investigating subgroup effects within a meta-analysis. It was shown that, even in the absence of ecological fallacy, there is significantly less bias and more precision when IPD is used over AD. Among models using IPD, there was less bias, better coverage, and higher precision in those that modelled the between-trial variation in interaction effects (when one existed), especially when a small number of trials was analysed, and thus should be given precedence whenever possible.

## Supplementary information

**Additional file 1 :** **Appendix A.** Code used to generate the simulated datasets. **Appendix B.** Performance measures. **Appendix C.** Results of the simulation study assuming a between-trial variance of 0.5 in the interaction effect. **Appendix D.** Results of the simulation study assuming a between-trial variance of 0.005 in the interaction effect. **Appendix E.** Results of the simulation study assuming a null between-trial variance in the interaction effect.

## Data Availability

Codes used to generate the simulated dataset are presented in the Additional file 1.

## Abbreviations

AD: Aggregate data
IPD: Individual patient data
RCT: Randomized clinical trial

## Footnotes

1 The actual simulated VAS values were not restricted within 0 and 10 to maintain a near normal shape of *y*_*ij*_ to ensure our results were generalizable to analyses assuming a Gaussian distribution.
