# Supplementary material for: A comparison of the statistical performance of different meta-analysis models for the synthesis of subgroup effects from randomized clinical trials
Source: BMC Med Res Methodol. 2019 Oct 26;19:198. doi: 10.1186/s12874-019-0831-8 (PMC6815379; doi:10.1186/s12874-019-0831-8)
Supplement: Supplementary file 1 — Additional file 1 : Appendix A. Code used to generate the simulated datasets. Appendix B. Performance measures. Appendix C. Results of the simulation study assuming a between-trial variance of 0.5 in the interaction effect. Appendix D. Results of the simulation study assuming a between-trial variance of 0.005 in the interaction effect. Appendix E. Results of the simulation study assuming a null between-trial variance in the interaction effect. [file 12874_2019_831_MOESM1_ESM.docx]

**APPENDICES**

**Contents**

[Appendix A. Code used to generate the simulated datasets. 2](#_Toc14186993)

[Appendix B. Performance measures. 7](#_Toc14186994)

[Appendix C. Results of the simulation study assuming a between-trial variance of 0.5 in the interaction effect. 10](#_Toc14186995)

[Appendix D. Results of the simulation study assuming a between-trial variance of 0.005 in the interaction effect. 14](#_Toc14186996)

[Appendix E. Results of the simulation study assuming a null between-trial variance in the interaction effect. 18](#_Toc14186997)

# Appendix A. Code used to generate the simulated datasets.

/* clear any data and programs in memory */

clear all

/* Define program */

cap program drop ipdsim

program define ipdsim, rclass

syntax [, NPLARGELOW(real 300) NPLARGEHIGH(real 500) NPSMALLLOW(real 50) NPSMALLHIGH(real 100) PSMALL(real 0.2) NT(real 10) ALLRAT(real 1) EFFECT(real -1) TAU2(real 0.5) INTERACT(real 0.1) TAUINTER(real 0.05) COVEFFECT(real 0.05)]

foreach x of numlist 1/`nt' {

********************************************************

preserve

clear

local nsmall=`nt'*`psmall'

if `nsmall'>=`x' {

local npsmall = round(runiform(`npsmalllow',`npsmallhigh'))

set obs `npsmall'

}

if `nsmall'<`x' {

local nplarge = round(runiform(`nplargelow',`nplargehigh'))

set obs `nplarge'

}

gen person = _n

gen trialid= `x'

*Generate Toeplitz 1 correlation matrix with structured errors (stronger correlations between observations that are closer together)

matrix V = ( 1.0, 0.8 \ ///

0.8, 1.0 )

matrix list V

*Generate matrix with mean values of random-error at patient level

matrix M = (0 \ 0)

*Generate matrix with standard deviation values of random error

matrix SD = (0.5 \ 0.2)

matrix list M

*Use the drawnorm command to generate random-effects intercept variable u_0 & random-effects slope variable u_1 that have a correlation of 0.8

drawnorm u_0 u_1, means(M) corr(V) sd(SD)

list in 1/5, noobs clean

sum u*

correlate u_0 u_1

local mean=rnormal(67,4.5)

local sd=rnormal(7,1)

gen age = rnormal(`mean',`sd')

count

local patcon=r(N)/(1+`allrat')

gen tx = cond(_n<=`patcon',0,1)

egen agecat=cut(age), at(40,50,60,70,80,90,100)

*Center age around the mean so that the intercept has a meaning

sum age

gen ageC=age-r(mean)

gen normal=rnormal(`effect',sqrt(`tau2'))

sum normal if _n==1

gen tx2=r(mean)

drop normal

gen normal=rnormal(`interact',sqrt(`tauinter'))

sum normal if _n==1

gen inter2=r(mean)

drop normal

*Mean baseline VAS can vary between trials

gen normal=rnormal(6,0.5)

sum normal if _n==`x'

gen intercept=r(mean)

drop normal

generate vas = intercept + tx2*tx + `coveffect'*age + inter2*age*tx + u_0 + u_1*tx

save "$dd\trial`x'", replace

restore

}

clear

foreach x of numlist 1/`nt' {

append using "$dd\trial`x'"

}

end

/* Define program */

cap program drop adsim

program define adsim, rclass

qui{

gen trialid2=.

gen npat_exp=.

gen npat_con=.

gen vas_mean_exp=.

gen vas_sd_exp=.

gen vas_mean_con=.

gen vas_sd_con=.

gen age_mean=.

gen age_sd=.

}

qui unique trialid

local nt=r(sum)

local l = 1

foreach x of numlist 1/`nt' {

qui {

replace trialid2=`x' in `l'

sum vas if trialid==`x' & tx==1

replace vas_mean_exp=r(mean) in `l'

replace vas_sd_exp=r(sd) in `l'

replace npat_exp=r(N) in `l'

sum vas if trialid==`x' & tx==0

replace vas_mean_con=r(mean) in `l'

replace vas_sd_con=r(sd) in `l'

replace npat_con=r(N) in `l'

sum age if trialid==`x'

replace age_mean=r(mean) in `l'

replace age_sd=r(sd) in `l'

local l = `l'+1

}

}

keep trialid2-age_sd

drop if trialid2==.

end

/* Define program */

cap program drop ipdsim2

program define ipdsim2, rclass

syntax [, NPLARGELOW2(real 300) NPLARGEHIGH2(real 500) NPSMALLLOW2(real 50) NPSMALLHIGH2(real 100) PSMALL2(real 0.2) NT2(real 10) ALLRAT2(real 1) EFFECT2(real -1) TAU22(real 0.5) INTERACT2(real 0.1) TAUINTER2(real 0.05) COVEFFECT2(real 0.05)]

ipdsim, nt(`nt2') psmall(`psmall2') nplargelow(`nplargelow2') nplargehigh(`nplargehigh2') npsmalllow(`npsmalllow2') npsmallhigh(`npsmallhigh2') allrat(`allrat2') effect(`effect2') tau2(`tau22') interact(`interact2') tauinter(`tauinter2') coveffect(`coveffect2')

egen id=group(person trialid)

gen ageCXtx=ageC*tx

gen ageXtx=age*tx

gen age_mean0=.

sum trialid

local maxid=r(max)

foreach id of numlist 1/`maxid' {

sum age if trialid==`id'

replace age_mean0=r(mean) if trialid==`id'

}

mixed vas tx##c.ageC tx##c.age_mean0 || trialid:tx || trialid:ageCXtx , covariance(exchangeable) robust , stddev difficult iterate(100)

matrix a=r(table)

matrix list a

return scalar x_ipd1 = a[1,5]

return scalar se_ipd1 = a[2,5]

return scalar p_ipd1 = a[4,5]

return scalar lci_ipd1 = a[5,5]

return scalar uci_ipd1 = a[6,5]

mixed vas tx##c.ageC tx##c.age_mean0 || trialid:ageCXtx , covariance(exchangeable) robust , stddev difficult iterate(100)

matrix a=r(table)

matrix list a

return scalar x_ipd2 = a[1,5]

return scalar se_ipd2 = a[2,5]

return scalar p_ipd2 = a[4,5]

return scalar lci_ipd2 = a[5,5]

return scalar uci_ipd2 = a[6,5]

mixed vas tx##c.ageC tx##c.age_mean0 || trialid:tx, covariance(exchangeable) robust , stddev difficult iterate(100)

matrix a=r(table)

matrix list a

return scalar x_ipd3 = a[1,5]

return scalar se_ipd3 = a[2,5]

return scalar p_ipd3 = a[4,5]

return scalar lci_ipd3 = a[5,5]

return scalar uci_ipd3 = a[6,5]

mixed vas tx##c.ageC || trialid:tx || trialid:ageCXtx, covariance(exchangeable) robust , stddev difficult iterate(100)

matrix a=r(table)

matrix list a

return scalar x_ipd4 = a[1,5]

return scalar se_ipd4 = a[2,5]

return scalar p_ipd4 = a[4,5]

return scalar lci_ipd4 = a[5,5]

return scalar uci_ipd4 = a[6,5]

mixed vas tx##c.ageC || trialid:ageCXtx, covariance(exchangeable) robust , stddev difficult iterate(100)

matrix a=r(table)

matrix list a

return scalar x_ipd5 = a[1,5]

return scalar se_ipd5 = a[2,5]

return scalar p_ipd5 = a[4,5]

return scalar lci_ipd5 = a[5,5]

return scalar uci_ipd5 = a[6,5]

mixed vas tx##c.ageC || trialid:tx, covariance(exchangeable) robust , stddev difficult iterate(100)

matrix a=r(table)

matrix list a

return scalar x_ipd6 = a[1,5]

return scalar se_ipd6 = a[2,5]

return scalar p_ipd6 = a[4,5]

return scalar lci_ipd6 = a[5,5]

return scalar uci_ipd6 = a[6,5]

adsim

gen diff= vas_mean_exp-vas_mean_con

gen diff_se=sqrt((vas_sd_exp/sqrt(npat_exp))^2+(vas_sd_con/sqrt(npat_con)^2))

metareg diff age_mean , wsse(diff_se)

matrix b=r(table)

matrix list b

return scalar x_ad = b[1,1]

return scalar se_ad = b[2,1]

return scalar p_ad = b[4,1]

return scalar lci_ad = b[5,1]

return scalar uci_ad = b[6,1]

end

clear

set seed 1234

timer clear

timer on 1

*set trace on

simulate x_ipd1=r(x_ipd1) se_ipd1=r(se_ipd1) p_ipd1=r(p_ipd1) lci_ipd1=r(lci_ipd1) uci_ipd1=r(uci_ipd1) x_ipd2=r(x_ipd2) se_ipd2=r(se_ipd2) p_ipd2=r(p_ipd2) lci_ipd2=r(lci_ipd2) uci_ipd2=r(uci_ipd2) ///

x_ipd3=r(x_ipd3) se_ipd3=r(se_ipd3) p_ipd3=r(p_ipd3) lci_ipd3=r(lci_ipd3) uci_ipd3=r(uci_ipd3) x_ipd4=r(x_ipd4) se_ipd4=r(se_ipd4) p_ipd4=r(p_ipd4) lci_ipd4=r(lci_ipd4) uci_ipd4=r(uci_ipd4) ///

x_ipd5=r(x_ipd5) se_ipd5=r(se_ipd5) p_ipd5=r(p_ipd5) lci_ipd5=r(lci_ipd5) uci_ipd5=r(uci_ipd5) x_ipd6=r(x_ipd6) se_ipd6=r(se_ipd6) p_ipd6=r(p_ipd6) lci_ipd6=r(lci_ipd6) uci_ipd6=r(uci_ipd6) ///

x_ad=r(x_ad) se_ad=r(se_ad) p_ad=r(p_ad) lci_ad=r(lci_ad) uci_ad=r(uci_ad), reps(2000): ///

ipdsim2, nt2(16) psmall2(0.5) npsmalllow2(30) npsmallhigh2(199) nplargelow2(200) nplargehigh2(400) allrat2(1) effect2(-1) tau22(0.0625) interact2(-0.01) tauinter2(0.05) coveffect2(0.05)

# Appendix B. Performance measures.

The current simulation study compares the performance of different models using the recommendations from both Morris et al and Burton et al. (13, 14). These performance measures are explained here.

*Bias* is our main performance measure of interest. It quantifies the difference between the average of the estimated interaction effect $\hat{\theta}$ acrossall n_sim_ simulations and the estimand *θ*, and it is calculated as follows:

$Bias=\frac{\sum_{i=1}^{n_{sim}} \hat{\theta}_{i}-\theta}{n_{sim}}$ (3.1)

${MCSE}_{Bias}=\sqrt{\frac{\sum_{i=1}^{n_{sim}} {(\hat{\theta}_{i}-\theta)}^{2}}{n_{sim}-1}}$ (3.2)

*Coverage* is the probability that a confidence interval contains the estimand *θ*. Undercoverage may occur in the presence of bias, if the model standard error is smaller than the empirical standard error, if $\hat{\theta}$ is not normally distributed and the confidence intervals assumed normality, or if the estimated variance of $\hat{\theta}_{i}$ is too variable. Overcoverage occurs if the model standard error is larger than the empirical standard error. Appropriate coverage occurs when coverage equals 100(1-*α*)%. Because in the present simulation *α*=0.05, appropriate coverage is 95%. Coverage is calculated as follows:

$Coverage=\frac{\sum_{i=1}^{n_{sim}} 1(\hat{\theta}_{low,i}\leq\theta\leq\hat{\theta}_{upp,i})}{n_{sim}}$ (3.3)

${MCSE}_{Coverage}=\sqrt{\frac{Coverage(1-Coverage)}{n_{sim}}}$ (3.4)

*Mean squared error* is the sum of the squared bias and variance of $\hat{\theta}$, and thus provides the interpretation of bias and variance of $\hat{\theta}$ in one single estimate. Higher values of the mean squared error indicate that $\hat{\theta}$ is biased, imprecise, or both. By providing one single estimate that incorporates both bias and precision, it facilitates the comparison of models regarding their performance. It is calculated as follows:

$Mean squared error=\frac{\sum_{i=1}^{n_{sim}} {(\hat{\theta}_{i}-\theta)}^{2}}{n_{sim}}$ (3.5)

${MCSE}_{Mean squared error}=\sqrt{\frac{\sum_{i=1}^{n_{sim}} {[\left( \hat{\theta}_{i}-\theta\right)^{2}-Mean squared error]}^{2}}{n_{sim}(n_{sim}-1)}}$ (3.6)

*Empirical SE* estimates the standard deviation of the estimated effect $\hat{\theta}$ over n_sim_ repetitions. As shown below, it only depends on the estimated effect $\hat{\theta}$ and its average across all repetitions $\bar{\theta}$:

$Empirical SE=\sqrt{\frac{\sum_{i=1}^{n_{sim}} {(\hat{\theta}_{i}-\bar{\theta})}^{2}}{n_{sim}-1}}$ (3.7)

${MCSE}_{Empirical SE}=\frac{Empirical SE}{\sqrt{2(n_{sim}-1)}}$ (3.8)

*Model SE* is the average estimated standard error of $\hat{\theta}$, which is calculated as follows:

$Model SE=\sqrt{\frac{\sum_{i=1}^{n_{sim}} \hat{Var}( \hat{\theta}_{i})}{n_{sim}}}$ (3.9)

${MCSE}_{Model SE}\approx\sqrt{\frac{Var[\hat{Var}\left( \hat{\theta}_{i} \right)]}{4n_{sim} X \hat{{Model SE}^{2}}}}$ (3.10)

*Relative % error in model SE*, is the percentage difference between the model SE and the empirical SE. The model SE is an estimation of the empirical SE, so when the model SE is smaller or larger than the empirical SE, it indicates that the model over- or underestimates precision, respectively. It is calculated as follows:

$Relative \% error in model SE=100\left( \frac{Model SE}{Empirical SE}-1 \right)$ (3.11)

${MCSE}_{Relative \% error in model SE}=100\left( \frac{Model SE}{Empirical SE} \right)\sqrt{\frac{Var[\hat{Var}\left( \hat{\theta}_{i} \right)]}{4n_{sim} X \hat{{Model SE}^{4}}}+\frac{1}{2(n-1)}}$ (3.12)

**Visual assessment of coverage using zip plots**

Zip plots are used to display the performance of statistical models in terms of coverage (14). These plots show on the y axis the centile-ranking of the confidence intervals according to their statistical significance against the simulated interaction effect *θ*. The x axis shows the actual values of the confidence intervals. Intervals that do not include *θ*, and thus have a p-value <0.05, are coloured black, while confidence intervals that include *θ* are coloured yellow. The red vertical line shows *θ*. In these graphs, an appropriate coverage is seen if the black confidence intervals do not cross the red line, and the red line is completely covered by the yellow confidence intervals.

#

# Appendix C. Results of the simulation study assuming a between-trial variance of 0.5 in the interaction effect.

**Appendix Table C.1.** Bias in the interaction effect between treatment effect and age in each of the models.

| **No. of trials** | **Model 1** | **Model 2** | **Model 3** | **Model 4** | **Model 5** | **Model 6** | **Model 7** |
| --- | --- | --- | --- | --- | --- | --- | --- |
| 6 | -0.00235 (0.28800) | -0.00300 (0.30155) | 0.00355 (0.33081) | -0.00235 (0.28800) | -0.00307 (0.30027) | 0.00355 (0.33081) | -0.03294 (6.25693) |
| 10 | 0.00301 (0.23058) | 0.00316 (0.24246) | 0.00170 (0.26810) | 0.00301 (0.23058) | 0.00221 (0.24181) | 0.00170 (0.26810) | 0.05127 (3.93329) |
| 16 | 0.00490 (0.17475) | 0.00489 (0.18774) | 0.00490 (0.20707) | 0.00490 (0.17475) | 0.00454 (0.18734) | 0.00490 (0.20707) | 0.07231 (2.83858) |
| 20 | -0.00551 (0.15532) | -0.00776 (0.16350) | -0.00639 (0.18401) | -0.00551 (0.15532) | -0.00787 (0.16352) | -0.00639 (0.18401) | -0.03089 (2.57765) |
| 26 | 0.00205 (0.14012) | 0.00134 (0.14627) | 0.00475 (0.16543) | 0.00205 (0.14012) | 0.00103 (0.14669) | 0.00475 (0.16543) | 0.00022 (2.26687) |
| 30 | -0.00425 (0.13138) | -0.00414 (0.13876) | -0.00361 (0.15076) | -0.00425 (0.13138) | -0.00422 (0.13852) | -0.00361 (0.15076) | -0.02953 (2.00626) |
| 40 | 0.00303 (0.11308) | 0.00349 (0.11939) | 0.00272 (0.13181) | 0.00303 (0.11308) | 0.00342 (0.11922) | 0.00272 (0.13181) | 0.07811 (1.72701) |
| 50 | -0.00175 (0.09910) | -0.00274 (0.10264) | -0.00221 (0.11673) | -0.00175 (0.09910) | -0.00284 (0.10248) | -0.00221 (0.11673) | -0.01012 (1.55867) |

Values in brackets are Monte Carlo standard errors

**Appendix Table C.2.** Observed coverage of the interaction effect between treatment effect and age in each of the models, with a nominal coverage of 95%.

| **No. of trials** | **Model 1** | **Model 2** | **Model 3** | **Model 4** | **Model 5** | **Model 6** | **Model 7** |
| --- | --- | --- | --- | --- | --- | --- | --- |
| 6 | 0.90250 (0.00663) | 0.89050 (0.00698) | 0.84900 (0.00801) | 0.90250 (0.00663) | 0.89350 (0.00690) | 0.84900 (0.00801) | 0.94150 (0.00525) |
| 10 | 0.90950 (0.00642) | 0.90400 (0.00659) | 0.87250 (0.00746) | 0.90950 (0.00642) | 0.90700 (0.00649) | 0.87250 (0.00746) | 0.95250 (0.00476) |
| 16 | 0.93600 (0.00547) | 0.93500 (0.00551) | 0.91050 (0.00638) | 0.93600 (0.00547) | 0.93450 (0.00553) | 0.91050 (0.00638) | 0.95450 (0.00466) |
| 20 | 0.94150 (0.00525) | 0.93800 (0.00539) | 0.91700 (0.00617) | 0.94150 (0.00525) | 0.93650 (0.00545) | 0.91700 (0.00617) | 0.95200 (0.00478) |
| 26 | 0.93150 (0.00565) | 0.93500 (0.00551) | 0.91950 (0.00608) | 0.93150 (0.00565) | 0.93200 (0.00563) | 0.91950 (0.00608) | 0.93550 (0.00549) |
| 30 | 0.94550 (0.00508) | 0.93550 (0.00549) | 0.93200 (0.00563) | 0.94550 (0.00508) | 0.93650 (0.00545) | 0.93200 (0.00563) | 0.95000 (0.00487) |
| 40 | 0.94150 (0.00525) | 0.93700 (0.00543) | 0.93100 (0.00567) | 0.94150 (0.00525) | 0.93850 (0.00537) | 0.93100 (0.00567) | 0.94850 (0.00494) |
| 50 | 0.94600 (0.00505) | 0.95100 (0.00483) | 0.94000 (0.00531) | 0.94600 (0.00505) | 0.94950 (0.00490) | 0.94000 (0.00531) | 0.94750 (0.00499) |

Values in brackets are Monte Carlo standard errors

**Appendix Table C.3**. Standard error of the interaction effect between treatment effect and age in each of the models.

| **No. of trials** | **Performance Measure** | **Model 1** | **Model 2** | **Model 3** | **Model 4** | **Model 5** | **Model 6** | **Model 7** |
| --- | --- | --- | --- | --- | --- | --- | --- | --- |
| 6 | Empirical SE | 0.28799 (0.00455) | 0.30153 (0.00477) | 0.33079 (0.00523) | 0.28799 (0.00455) | 0.30026 (0.00475) | 0.33079 (0.00523) | 6.25684 (0.09895) |
|  | Model SE | 0.28931 (0.33213) | 0.30156 (0.42745) | 0.30415 (0.52019) | 0.28931 (0.33213) | 0.30275 (0.45837) | 0.30415 (0.52019) | 6.10213 (7.73e+03) |
|  | Relative error in model SE | 0.45741 (1.73170) | 0.00934 (1.76329) | -8.05329 (1.68436) | 0.45742 (1.73170) | 0.83005 (1.79902) | -8.05330 (1.68436) | -2.47268 (2.26459) |
| 10 | Empirical SE | 0.23056 (0.00365) | 0.24244 (0.00383) | 0.26810 (0.00424) | 0.23056 (0.00365) | 0.24180 (0.00382) | 0.26810 (0.00424) | 3.93296 (0.06220) |
|  | Model SE | 0.22345 (0.12244) | 0.23524 (0.18411) | 0.24761 (0.23164) | 0.22345 (0.12244) | 0.23520 (0.18166) | 0.24761 (0.23164) | 3.98098 (1.23e+03) |
|  | Relative error in model SE | -3.08274 (1.62241) | -2.97261 (1.68095) | -7.64026 (1.62176) | -3.08231 (1.62242) | -2.72678 (1.68160) | -7.64025 (1.62176) | 1.22085 (1.88197) |
| 16 | Empirical SE | 0.17469 (0.00276) | 0.18768 (0.00297) | 0.20702 (0.00327) | 0.17469 (0.00276) | 0.18729 (0.00296) | 0.20702 (0.00327) | 2.83766 (0.04488) |
|  | Model SE | 0.17676 (0.04584) | 0.18646 (0.08780) | 0.20170 (0.10254) | 0.17676 (0.04584) | 0.18648 (0.08724) | 0.20170 (0.10254) | 2.90742 (323.32309) |
|  | Relative error in model SE | 1.18887 (1.65452) | -0.64816 (1.70925) | -2.56800 (1.65682) | 1.18918 (1.65453) | -0.42910 (1.71126) | -2.56800 (1.65682) | 2.45821 (1.75498) |
| 20 | Empirical SE | 0.15522 (0.00245) | 0.16331 (0.00258) | 0.18390 (0.00291) | 0.15522 (0.00245) | 0.16333 (0.00258) | 0.18390 (0.00291) | 2.57746 (0.04076) |
|  | Model SE | 0.15753 (0.02852) | 0.16635 (0.04567) | 0.18085 (0.06448) | 0.15753 (0.02852) | 0.16616 (0.04453) | 0.18085 (0.06448) | 2.51723 (183.07433) |
|  | Relative error in model SE | 1.48896 (1.64721) | 1.86014 (1.68835) | -1.65989 (1.64506) | 1.48874 (1.64721) | 1.73499 (1.68302) | -1.65988 (1.64506) | -2.33676 (1.64312) |
| 26 | Empirical SE | 0.14011 (0.00222) | 0.14627 (0.00231) | 0.16537 (0.00262) | 0.14011 (0.00222) | 0.14668 (0.00232) | 0.16537 (0.00262) | 2.26687 (0.03585) |
|  | Model SE | 0.13953 (0.01723) | 0.14623 (0.02447) | 0.16054 (0.03924) | 0.13953 (0.01723) | 0.14623 (0.02455) | 0.16054 (0.03924) | 2.18596 (99.16687) |
|  | Relative error in model SE | -0.41344 (1.60634) | -0.02708 (1.62877) | -2.92050 (1.60287) | -0.41338 (1.60634) | -0.30895 (1.62447) | -2.92050 (1.60287) | -3.56926 (1.59230) |
| 30 | Empirical SE | 0.13131 (0.00208) | 0.13870 (0.00219) | 0.15072 (0.00238) | 0.13131 (0.00208) | 0.13845 (0.00219) | 0.15072 (0.00238) | 2.00604 (0.03173) |
|  | Model SE | 0.12851 (0.01212) | 0.13466 (0.01702) | 0.14874 (0.02836) | 0.12851 (0.01212) | 0.13453 (0.01714) | 0.14874 (0.02836) | 2.00041 (70.04490) |
|  | Relative error in model SE | -2.13529 (1.57278) | -2.91189 (1.57235) | -1.31172 (1.61768) | -2.13528 (1.57278) | -2.83450 (1.57432) | -1.31172 (1.61768) | -0.28058 (1.63633) |
| 40 | Empirical SE | 0.11304 (0.00179) | 0.11934 (0.00189) | 0.13178 (0.00208) | 0.11304 (0.00179) | 0.11917 (0.00188) | 0.13178 (0.00208) | 1.72524 (0.02729) |
|  | Model SE | 0.11169 (0.00688) | 0.11760 (0.01090) | 0.12973 (0.01713) | 0.11169 (0.00688) | 0.11751 (0.01077) | 0.12973 (0.01713) | 1.72278 (38.81337) |
|  | Relative error in model SE | -1.20128 (1.58146) | -1.45558 (1.59314) | -1.55654 (1.60407) | -1.20142 (1.58145) | -1.39897 (1.59338) | -1.55654 (1.60407) | -0.14266 (1.62412) |
| 50 | Empirical SE | 0.09909 (0.00157) | 0.10260 (0.00162) | 0.11671 (0.00185) | 0.09909 (0.00157) | 0.10244 (0.00162) | 0.11671 (0.00185) | 1.55863 (0.02465) |
|  | Model SE | 0.10002 (0.00442) | 0.10488 (0.00647) | 0.11602 (0.01072) | 0.10002 (0.00442) | 0.10481 (0.00647) | 0.11602 (0.01072) | 1.52903 (23.55597) |
|  | Relative error in model SE | 0.93589 (1.61182) | 2.22239 (1.64193) | -0.59224 (1.60877) | 0.93585 (1.61182) | 2.30875 (1.64341) | -0.59224 (1.60877) | -1.89922 (1.58481) |

Values in brackets are Monte Carlo standard errors

**Appendix Table C.4.** Mean squared error of the interaction effect between treatment effect and age in each of the models.

| **No. of trials** | **Model 1** | **Model 2** | **Model 3** | **Model 4** | **Model 5** | **Model 6** | **Model 7** |  |
| --- | --- | --- | --- | --- | --- | --- | --- | --- |
| 6 | 0.08291 (0.00261) | 0.09089 (0.00293) | 0.10938 (0.00341) | 0.08290 (0.00261) | 0.09012 (0.00295) | 0.10938 (0.00341) | 39.12962 (1.90653) | |
| 10 | 0.05314 (0.00164) | 0.05876 (0.00192) | 0.07184 (0.00215) | 0.05314 (0.00164) | 0.05844 (0.00189) | 0.07184 (0.00215) | 15.46307 (0.58252) | |
| 16 | 0.03052 (0.00095) | 0.03523 (0.00125) | 0.04286 (0.00138) | 0.03052 (0.00095) | 0.03508 (0.00124) | 0.04286 (0.00138) | 8.05353 (0.27280) | |
| 20 | 0.02411 (0.00077) | 0.02672 (0.00085) | 0.03384 (0.00111) | 0.02411 (0.00077) | 0.02672 (0.00085) | 0.03384 (0.00111) | 6.64095 (0.23704) | |
| 26 | 0.01962 (0.00061) | 0.02138 (0.00066) | 0.02735 (0.00086) | 0.01962 (0.00061) | 0.02151 (0.00066) | 0.02735 (0.00086) | 5.13611 (0.16868) | |
| 30 | 0.01725 (0.00057) | 0.01924 (0.00062) | 0.02272 (0.00074) | 0.01725 (0.00057) | 0.01918 (0.00062) | 0.02272 (0.00074) | 4.02306 (0.13166) | |
| 40 | 0.01278 (0.00040) | 0.01425 (0.00044) | 0.01737 (0.00055) | 0.01278 (0.00040) | 0.01421 (0.00044) | 0.01737 (0.00055) | 2.98106 (0.10338) | |
| 50 | 0.00982 (0.00031) | 0.01053 (0.00033) | 0.01362 (0.00043) | 0.00982 (0.00031) | 0.01050 (0.00033) | 0.01362 (0.00043) | 2.42823 (0.07661) | |

Values in brackets are Monte Carlo standard errors

# Appendix D. Results of the simulation study assuming a between-trial variance of 0.005 in the interaction effect.

**Appendix Table D.1.** Bias in the interaction effect between treatment effect and age in each of the models.

| **No. of trials** | **Model 1** | **Model 2** | **Model 3** | **Model 4** | **Model 5** | **Model 6** | **Model 7** |
| --- | --- | --- | --- | --- | --- | --- | --- |
| 6 | 0.00045 (0.02933) | -0.00013 (0.03070) | 0.00097 (0.03294) | 0.00045 (0.02934) | 0.00001 (0.03077) | 0.00097 (0.03294) | -0.02082 (0.59415) |
| 10 | -0.00031 (0.02280) | -0.00024 (0.02380) | -0.00024 (0.02608) | -0.00031 (0.02280) | -0.00021 (0.02381) | -0.00024 (0.02608) | 0.00403 (0.38782) |
| 16 | -0.00005 (0.01773) | -0.00020 (0.01853) | -0.00030 (0.02101) | -0.00005 (0.01773) | -0.00018 (0.01854) | -0.00030 (0.02101) | 0.00559 (0.29456) |
| 20 | -0.00019 (0.01615) | -0.00036 (0.01694) | -0.00040 (0.01883) | -0.00019 (0.01615) | -0.00035 (0.01692) | -0.00040 (0.01883) | -0.00735 (0.25938) |
| 26 | -0.00018 (0.01385) | -0.00027 (0.01459) | 0.00003 (0.01634) | -0.00018 (0.01385) | -0.00026 (0.01457) | 0.00003 (0.01634) | -0.00336 (0.21528) |
| 30 | -0.00005 (0.01323) | 0.00016 (0.01394) | 0.00007 (0.01559) | -0.00005 (0.01323) | 0.00015 (0.01392) | 0.00007 (0.01559) | 0.00320 (0.19851) |
| 40 | 0.00012 (0.01128) | 0.00025 (0.01192) | 0.00016 (0.01335) | 0.00012 (0.01128) | 0.00025 (0.01191) | 0.00016 (0.01335) | 0.00597 (0.17468) |
| 50 | -0.00005 (0.01029) | -0.00003 (0.01080) | -0.00005 (0.01213) | -0.00005 (0.01029) | -0.00002 (0.01081) | -0.00005 (0.01213) | 0.00005 (0.15290) |

Values in brackets are Monte Carlo standard errors

**Appendix Table D.2.** Observed coverage of the interaction effect between treatment effect and age in each of the models, with a nominal coverage of 95%.

| **No. of trials** | **Model 1** | **Model 2** | **Model 3** | **Model 4** | **Model 5** | **Model 6** | **Model 7** |
| --- | --- | --- | --- | --- | --- | --- | --- |
| 6 | 0.89400 (0.00688) | 0.88500 (0.00713) | 0.83850 (0.00823) | 0.89450 (0.00687) | 0.88550 (0.00712) | 0.83850 (0.00823) | 0.94350 (0.00516) |
| 10 | 0.92250 (0.00598) | 0.92100 (0.00603) | 0.88950 (0.00701) | 0.92250 (0.00598) | 0.92150 (0.00601) | 0.88950 (0.00701) | 0.95250 (0.00476) |
| 16 | 0.92850 (0.00576) | 0.92600 (0.00585) | 0.90800 (0.00646) | 0.92900 (0.00574) | 0.92700 (0.00582) | 0.90800 (0.00646) | 0.94900 (0.00492) |
| 20 | 0.93350 (0.00557) | 0.93900 (0.00535) | 0.92350 (0.00594) | 0.93400 (0.00555) | 0.93450 (0.00553) | 0.92350 (0.00594) | 0.94050 (0.00529) |
| 26 | 0.94150 (0.00525) | 0.94300 (0.00518) | 0.93250 (0.00561) | 0.94150 (0.00525) | 0.94250 (0.00521) | 0.93250 (0.00561) | 0.95300 (0.00473) |
| 30 | 0.93150 (0.00565) | 0.93300 (0.00559) | 0.91750 (0.00615) | 0.93150 (0.00565) | 0.93400 (0.00555) | 0.91750 (0.00615) | 0.94900 (0.00492) |
| 40 | 0.94650 (0.00503) | 0.93800 (0.00539) | 0.93250 (0.00561) | 0.94650 (0.00503) | 0.93700 (0.00543) | 0.93250 (0.00561) | 0.94450 (0.00512) |
| 50 | 0.94700 (0.00501) | 0.94950 (0.00490) | 0.93400 (0.00555) | 0.94700 (0.00501) | 0.94900 (0.00492) | 0.93400 (0.00555) | 0.95400 (0.00468) |

Values in brackets are Monte Carlo standard errors

**Appendix Table D.3**. Standard error of the interaction effect between treatment effect and age in each of the models.

| **No. of trials** | **Performance Measure** | **Model 1** | **Model 2** | **Model 3** | **Model 4** | **Model 5** | **Model 6** | **Model 7** |
| --- | --- | --- | --- | --- | --- | --- | --- | --- |
| 6 | Empirical SE | 0.02933 (0.00046) | 0.03070 (0.00049) | 0.03292 (0.00052) | 0.02933 (0.00046) | 0.03077 (0.00049) | 0.03292 (0.00052) | 0.59379 (0.00939) |
|  | Model SE | 0.02939 (0.00035) | 0.03052 (0.00048) | 0.03062 (0.00054) | 0.02939 (0.00035) | 0.03064 (0.00048) | 0.03062 (0.00054) | 0.57591 (5.67284) |
|  | Relative error in model SE | 0.21599 (1.73231) | -0.57878 (1.78447) | -6.99770 (1.71063) | 0.20522 (1.73213) | -0.44198 (1.77814) | -6.99743 (1.71063) | -3.01137 (2.10408) |
| 10 | Empirical SE | 0.02280 (0.00036) | 0.02380 (0.00038) | 0.02608 (0.00041) | 0.02280 (0.00036) | 0.02381 (0.00038) | 0.02608 (0.00041) | 0.38780 (0.00613) |
|  | Model SE | 0.02279 (0.00013) | 0.02386 (0.00018) | 0.02484 (0.00022) | 0.02279 (0.00013) | 0.02386 (0.00018) | 0.02484 (0.00022) | 0.39141 (1.14392) |
|  | Relative error in model SE | -0.04128 (1.66858) | 0.24104 (1.71942) | -4.72879 (1.65378) | -0.03836 (1.66863) | 0.22176 (1.71568) | -4.72870 (1.65379) | 0.93045 (1.86410) |
| 16 | Empirical SE | 0.01773 (0.00028) | 0.01853 (0.00029) | 0.02101 (0.00033) | 0.01773 (0.00028) | 0.01854 (0.00029) | 0.02101 (0.00033) | 0.29451 (0.00466) |
|  | Model SE | 0.01802 (0.00005) | 0.01889 (0.00007) | 0.02035 (0.00010) | 0.01802 (0.00005) | 0.01884 (0.00006) | 0.02035 (0.00010) | 0.29172 (0.33472) |
|  | Relative error in model SE | 1.60206 (1.65962) | 1.94227 (1.68767) | -3.15016 (1.64412) | 1.60001 (1.65959) | 1.61261 (1.68032) | -3.15022 (1.64412) | -0.94713 (1.70294) |
| 20 | Empirical SE | 0.01615 (0.00026) | 0.01694 (0.00027) | 0.01883 (0.00030) | 0.01615 (0.00026) | 0.01692 (0.00027) | 0.01883 (0.00030) | 0.25928 (0.00410) |
|  | Model SE | 0.01610 (0.00003) | 0.01688 (0.00004) | 0.01825 (0.00007) | 0.01610 (0.00003) | 0.01688 (0.00004) | 0.01825 (0.00007) | 0.25604 (0.19445) |
|  | Relative error in model SE | -0.32841 (1.61531) | -0.35190 (1.63525) | -3.08075 (1.62203) | -0.33077 (1.61527) | -0.23172 (1.63832) | -3.08075 (1.62203) | -1.24860 (1.66324) |
| 26 | Empirical SE | 0.01384 (0.00022) | 0.01459 (0.00023) | 0.01634 (0.00026) | 0.01384 (0.00022) | 0.01457 (0.00023) | 0.01634 (0.00026) | 0.21526 (0.00340) |
|  | Model SE | 0.01418 (0.00002) | 0.01484 (0.00002) | 0.01616 (0.00004) | 0.01418 (0.00002) | 0.01483 (0.00002) | 0.01616 (0.00004) | 0.21955 (0.10121) |
|  | Relative error in model SE | 2.45488 (1.65234) | 1.73228 (1.65425) | -1.14071 (1.63553) | 2.45244 (1.65230) | 1.78828 (1.65417) | -1.14075 (1.63553) | 1.99534 (1.68520) |
| 30 | Empirical SE | 0.01323 (0.00021) | 0.01394 (0.00022) | 0.01559 (0.00025) | 0.01323 (0.00021) | 0.01392 (0.00022) | 0.01559 (0.00025) | 0.19848 (0.00314) |
|  | Model SE | 0.01311 (0.00001) | 0.01376 (0.00002) | 0.01493 (0.00003) | 0.01311 (0.00001) | 0.01375 (0.00002) | 0.01493 (0.00003) | 0.20080 (0.07310) |
|  | Relative error in model SE | -0.88428 (1.59466) | -1.29675 (1.60218) | -4.20743 (1.57406) | -0.88689 (1.59462) | -1.21491 (1.60373) | -4.20743 (1.57406) | 1.16428 (1.66387) |
| 40 | Empirical SE | 0.01128 (0.00018) | 0.01192 (0.00019) | 0.01335 (0.00021) | 0.01128 (0.00018) | 0.01191 (0.00019) | 0.01335 (0.00021) | 0.17457 (0.00276) |
|  | Model SE | 0.01142 (0.00001) | 0.01197 (0.00001) | 0.01307 (0.00002) | 0.01142 (0.00001) | 0.01196 (0.00001) | 0.01307 (0.00002) | 0.17241 (0.03921) |
|  | Relative error in model SE | 1.16792 (1.61984) | 0.41676 (1.62151) | -2.06127 (1.59353) | 1.16905 (1.61986) | 0.42910 (1.62146) | -2.06126 (1.59353) | -1.23803 (1.60700) |
| 50 | Empirical SE | 0.01029 (0.00016) | 0.01080 (0.00017) | 0.01213 (0.00019) | 0.01029 (0.00016) | 0.01081 (0.00017) | 0.01213 (0.00019) | 0.15290 (0.00242) |
|  | Model SE | 0.01019 (0.00000) | 0.01072 (0.00001) | 0.01172 (0.00001) | 0.01019 (0.00000) | 0.01072 (0.00001) | 0.01172 (0.00001) | 0.15271 (0.02392) |
|  | Relative error in model SE | -0.99075 (1.58176) | -0.73436 (1.59486) | -3.37463 (1.56358) | -0.99290 (1.58173) | -0.82841 (1.59363) | -3.37464 (1.56358) | -0.12523 (1.61479) |

Values in brackets are Monte Carlo standard errors

**Appendix Table D.4.** Mean squared error of the interaction effect between treatment effect and age in each of the models.

| **No. of trials** | **Model 1** | **Model 2** | **Model 3** | **Model 4** | **Model 5** | **Model 6** | **Model 7** |
| --- | --- | --- | --- | --- | --- | --- | --- |
| 6 | 0.00086 (0.00003) | 0.00094 (0.00003) | 0.00108 (0.00003) | 0.00086 (0.00003) | 0.00095 (0.00003) | 0.00108 (0.00003) | 0.35284 (0.01835) |
| 10 | 0.00052 (0.00002) | 0.00057 (0.00002) | 0.00068 (0.00002) | 0.00052 (0.00002) | 0.00057 (0.00002) | 0.00068 (0.00002) | 0.15033 (0.00560) |
| 16 | 0.00031 (0.00001) | 0.00034 (0.00001) | 0.00044 (0.00001) | 0.00031 (0.00001) | 0.00034 (0.00001) | 0.00044 (0.00001) | 0.08672 (0.00340) |
| 20 | 0.00026 (0.00001) | 0.00029 (0.00001) | 0.00035 (0.00001) | 0.00026 (0.00001) | 0.00029 (0.00001) | 0.00035 (0.00001) | 0.06725 (0.00231) |
| 26 | 0.00019 (0.00001) | 0.00021 (0.00001) | 0.00027 (0.00001) | 0.00019 (0.00001) | 0.00021 (0.00001) | 0.00027 (0.00001) | 0.04632 (0.00158) |
| 30 | 0.00017 (0.00001) | 0.00019 (0.00001) | 0.00024 (0.00001) | 0.00017 (0.00001) | 0.00019 (0.00001) | 0.00024 (0.00001) | 0.03939 (0.00133) |
| 40 | 0.00013 (0.00000) | 0.00014 (0.00000) | 0.00018 (0.00001) | 0.00013 (0.00000) | 0.00014 (0.00000) | 0.00018 (0.00001) | 0.03050 (0.00099) |
| 50 | 0.00011 (0.00000) | 0.00012 (0.00000) | 0.00015 (0.00000) | 0.00011 (0.00000) | 0.00012 (0.00000) | 0.00015 (0.00000) | 0.02337 (0.00077) |

Values in brackets are Monte Carlo standard errors

# Appendix E. Results of the simulation study assuming a null between-trial variance in the interaction effect.

**Appendix Table E.1.** Bias in the interaction effect between treatment effect and age in each of the models.

| **No. of trials** | **Model 1** | **Model 2** | **Model 3** | **Model 4** | **Model 5** | **Model 6** | **Model 7** |
| --- | --- | --- | --- | --- | --- | --- | --- |
| 6 | 0.00018 (0.00578) | 0.00017 (0.00583) | 0.00025 (0.00482) | 0.00018 (0.00581) | 0.00016 (0.00584) | 0.00025 (0.00482) | 0.00046 (0.03512) |
| 10 | 0.00004 (0.00459) | 0.00004 (0.00460) | -0.00003 (0.00377) | 0.00004 (0.00459) | 0.00003 (0.00460) | -0.00003 (0.00377) | -0.00005 (0.02306) |
| 16 | 0.00015 (0.00356) | 0.00015 (0.00358) | 0.00006 (0.00293) | 0.00015 (0.00356) | 0.00014 (0.00358) | 0.00006 (0.00293) | -0.00039 (0.01684) |
| 20 | -0.00007 (0.00313) | -0.00007 (0.00313) | -0.00008 (0.00260) | -0.00007 (0.00313) | -0.00007 (0.00313) | -0.00008 (0.00260) | -0.00019 (0.01455) |
| 26 | 0.00007 (0.00279) | 0.00007 (0.00281) | 0.00002 (0.00231) | 0.00007 (0.00279) | 0.00007 (0.00281) | 0.00002 (0.00231) | 0.00014 (0.01258) |
| 30 | 0.00006 (0.00262) | 0.00005 (0.00262) | 0.00004 (0.00214) | 0.00006 (0.00261) | 0.00005 (0.00262) | 0.00004 (0.00214) | 0.00021 (0.01143) |
| 40 | -0.00001 (0.00226) | -0.00001 (0.00227) | 0.00001 (0.00188) | -0.00000 (0.00226) | -0.00001 (0.00227) | 0.00001 (0.00188) | -0.00018 (0.00987) |
| 50 | -0.00002 (0.00199) | -0.00002 (0.00199) | -0.00002 (0.00165) | -0.00002 (0.00199) | -0.00002 (0.00199) | -0.00002 (0.00165) | 0.00012 (0.00881) |

Values in brackets are Monte Carlo standard errors

**Appendix Table E.2.** Observed coverage of the interaction effect between treatment effect and age in each of the models, with a nominal coverage of 95%.

| **No. of trials** | **Model 1** | **Model 2** | **Model 3** | **Model 4** | **Model 5** | **Model 6** | **Model 7** |
| --- | --- | --- | --- | --- | --- | --- | --- |
| 6 | 0.88650 (0.00709) | 0.88550 (0.00712) | 0.85900 (0.00778) | 0.88850 (0.00704) | 0.88600 (0.00711) | 0.85950 (0.00777) | 0.92600 (0.00585) |
| 10 | 0.91250 (0.00632) | 0.91300 (0.00630) | 0.90150 (0.00666) | 0.91250 (0.00632) | 0.91250 (0.00632) | 0.90200 (0.00665) | 0.94700 (0.00501) |
| 16 | 0.93500 (0.00551) | 0.93500 (0.00551) | 0.92350 (0.00594) | 0.93550 (0.00549) | 0.93550 (0.00549) | 0.92300 (0.00596) | 0.94650 (0.00503) |
| 20 | 0.95050 (0.00485) | 0.95000 (0.00487) | 0.93350 (0.00557) | 0.95000 (0.00487) | 0.95000 (0.00487) | 0.93350 (0.00557) | 0.94900 (0.00492) |
| 26 | 0.93900 (0.00535) | 0.93950 (0.00533) | 0.92500 (0.00589) | 0.93950 (0.00533) | 0.94050 (0.00529) | 0.92450 (0.00591) | 0.95150 (0.00480) |
| 30 | 0.94350 (0.00516) | 0.94600 (0.00505) | 0.93400 (0.00555) | 0.94450 (0.00512) | 0.94700 (0.00501) | 0.93400 (0.00555) | 0.95600 (0.00459) |
| 40 | 0.94150 (0.00525) | 0.94350 (0.00516) | 0.92750 (0.00580) | 0.94150 (0.00525) | 0.94400 (0.00514) | 0.92800 (0.00578) | 0.94550 (0.00508) |
| 50 | 0.94650 (0.00503) | 0.94450 (0.00512) | 0.93500 (0.00551) | 0.94700 (0.00501) | 0.94500 (0.00510) | 0.93550 (0.00549) | 0.94400 (0.00514) |

Values in brackets are Monte Carlo standard errors

**Appendix Table E.3**. Standard error of the interaction effect between treatment effect and age in each of the models.

| **No. of trials** | **Performance Measure** | **Model 1** | **Model 2** | **Model 3** | **Model 4** | **Model 5** | **Model 6** | **Model 7** |
| --- | --- | --- | --- | --- | --- | --- | --- | --- |
| 6 | Empirical SE | 0.00578 (0.00009) | 0.00583 (0.00009) | 0.00482 (0.00008) | 0.00581 (0.00009) | 0.00584 (0.00009) | 0.00482 (0.00008) | 0.03512 (0.00056) |
|  | Model SE | 0.00570 (0.00000) | 0.00573 (0.00000) | 0.00462 (0.00000) | 0.00572 (0.00000) | 0.00574 (0.00000) | 0.00462 (0.00000) | 0.03518 (0.00276) |
|  | Relative error in model SE | -1.37430 (1.78159) | -1.71894 (1.77381) | -4.10781 (1.71633) | -1.61492 (1.77820) | -1.75139 (1.77479) | -4.10501 (1.71653) | 0.17286 (3.54774) |
| 10 | Empirical SE | 0.00459 (0.00007) | 0.00460 (0.00007) | 0.00377 (0.00006) | 0.00459 (0.00007) | 0.00460 (0.00007) | 0.00377 (0.00006) | 0.02306 (0.00036) |
|  | Model SE | 0.00451 (0.00000) | 0.00453 (0.00000) | 0.00365 (0.00000) | 0.00451 (0.00000) | 0.00453 (0.00000) | 0.00365 (0.00000) | 0.02253 (0.00021) |
|  | Relative error in model SE | -1.78587 (1.75865) | -1.52782 (1.82733) | -3.07609 (1.64723) | -1.78009 (1.75228) | -1.54031 (1.81648) | -3.06983 (1.64728) | -2.29527 (1.78343) |
| 16 | Empirical SE | 0.00356 (0.00006) | 0.00357 (0.00006) | 0.00293 (0.00005) | 0.00356 (0.00006) | 0.00358 (0.00006) | 0.00293 (0.00005) | 0.01683 (0.00027) |
|  | Model SE | 0.00353 (0.00000) | 0.00354 (0.00000) | 0.00289 (0.00000) | 0.00353 (0.00000) | 0.00354 (0.00000) | 0.00289 (0.00000) | 0.01661 (0.00006) |
|  | Relative error in model SE | -0.82186 (1.65855) | -1.03660 (1.65600) | -1.24458 (1.63278) | -0.84151 (1.65894) | -1.09597 (1.65643) | -1.22688 (1.63306) | -1.32138 (1.67480) |
| 20 | Empirical SE | 0.00313 (0.00005) | 0.00313 (0.00005) | 0.00260 (0.00004) | 0.00313 (0.00005) | 0.00313 (0.00005) | 0.00260 (0.00004) | 0.01455 (0.00023) |
|  | Model SE | 0.00319 (0.00000) | 0.00320 (0.00000) | 0.00260 (0.00000) | 0.00319 (0.00000) | 0.00320 (0.00000) | 0.00260 (0.00000) | 0.01458 (0.00003) |
|  | Relative error in model SE | 1.93887 (1.68876) | 2.11067 (1.69222) | -0.02047 (1.63947) | 1.92501 (1.68882) | 2.07000 (1.69182) | -0.01139 (1.63963) | 0.19154 (1.68243) |
| 26 | Empirical SE | 0.00279 (0.00004) | 0.00280 (0.00004) | 0.00231 (0.00004) | 0.00279 (0.00004) | 0.00280 (0.00004) | 0.00231 (0.00004) | 0.01258 (0.00020) |
|  | Model SE | 0.00279 (0.00000) | 0.00280 (0.00000) | 0.00228 (0.00000) | 0.00279 (0.00000) | 0.00280 (0.00000) | 0.00228 (0.00000) | 0.01261 (0.00002) |
|  | Relative error in model SE | -0.03384 (1.63844) | -0.29374 (1.63576) | -1.13975 (1.60803) | -0.05417 (1.63811) | -0.30426 (1.63568) | -1.14326 (1.60796) | 0.18349 (1.65581) |
| 30 | Empirical SE | 0.00261 (0.00004) | 0.00262 (0.00004) | 0.00214 (0.00003) | 0.00261 (0.00004) | 0.00262 (0.00004) | 0.00214 (0.00003) | 0.01143 (0.00018) |
|  | Model SE | 0.00261 (0.00000) | 0.00262 (0.00000) | 0.00211 (0.00000) | 0.00261 (0.00000) | 0.00262 (0.00000) | 0.00211 (0.00000) | 0.01160 (0.00001) |
|  | Relative error in model SE | -0.08023 (1.63454) | 0.11908 (1.63830) | -1.53094 (1.59604) | -0.05068 (1.63509) | 0.15070 (1.63874) | -1.51533 (1.59631) | 1.45986 (1.66584) |
| 40 | Empirical SE | 0.00226 (0.00004) | 0.00227 (0.00004) | 0.00188 (0.00003) | 0.00226 (0.00004) | 0.00227 (0.00004) | 0.00188 (0.00003) | 0.00987 (0.00016) |
|  | Model SE | 0.00225 (0.00000) | 0.00226 (0.00000) | 0.00185 (0.00000) | 0.00225 (0.00000) | 0.00226 (0.00000) | 0.00185 (0.00000) | 0.00994 (0.00001) |
|  | Relative error in model SE | -0.29764 (1.60952) | -0.49222 (1.60624) | -1.70512 (1.58375) | -0.27178 (1.60996) | -0.44625 (1.60695) | -1.67873 (1.58418) | 0.69938 (1.63731) |
| 50 | Empirical SE | 0.00199 (0.00003) | 0.00199 (0.00003) | 0.00165 (0.00003) | 0.00199 (0.00003) | 0.00199 (0.00003) | 0.00165 (0.00003) | 0.00881 (0.00014) |
|  | Model SE | 0.00201 (0.00000) | 0.00201 (0.00000) | 0.00165 (0.00000) | 0.00201 (0.00000) | 0.00201 (0.00000) | 0.00165 (0.00000) | 0.00882 (0.00000) |
|  | Relative error in model SE | 1.10876 (1.62796) | 0.83568 (1.62309) | -0.44429 (1.59754) | 1.14158 (1.62858) | 0.90809 (1.62423) | -0.43854 (1.59762) | 0.05774 (1.61931) |

Values in brackets are Monte Carlo standard errors

**Appendix Table E.4.** Mean squared error of the interaction effect between treatment effect and age in each of the models.

| **No. of trials** | **Model 1** | **Model 2** | **Model 3** | **Model 4** | **Model 5** | **Model 6** | **Model 7** |
| --- | --- | --- | --- | --- | --- | --- | --- |
| 6 | 0.00003 (0.00000) | 0.00003 (0.00000) | 0.00002 (0.00000) | 0.00003 (0.00000) | 0.00003 (0.00000) | 0.00002 (0.00000) | 0.00123 (0.00006) |
| 10 | 0.00002 (0.00000) | 0.00002 (0.00000) | 0.00001 (0.00000) | 0.00002 (0.00000) | 0.00002 (0.00000) | 0.00001 (0.00000) | 0.00053 (0.00002) |
| 16 | 0.00001 (0.00000) | 0.00001 (0.00000) | 0.00001 (0.00000) | 0.00001 (0.00000) | 0.00001 (0.00000) | 0.00001 (0.00000) | 0.00028 (0.00001) |
| 20 | 0.00001 (0.00000) | 0.00001 (0.00000) | 0.00001 (0.00000) | 0.00001 (0.00000) | 0.00001 (0.00000) | 0.00001 (0.00000) | 0.00021 (0.00001) |
| 26 | 0.00001 (0.00000) | 0.00001 (0.00000) | 0.00001 (0.00000) | 0.00001 (0.00000) | 0.00001 (0.00000) | 0.00001 (0.00000) | 0.00016 (0.00001) |
| 30 | 0.00001 (0.00000) | 0.00001 (0.00000) | 0.00000 (0.00000) | 0.00001 (0.00000) | 0.00001 (0.00000) | 0.00000 (0.00000) | 0.00013 (0.00000) |
| 40 | 0.00001 (0.00000) | 0.00001 (0.00000) | 0.00000 (0.00000) | 0.00001 (0.00000) | 0.00001 (0.00000) | 0.00000 (0.00000) | 0.00010 (0.00000) |
| 50 | 0.00000 (0.00000) | 0.00000 (0.00000) | 0.00000 (0.00000) | 0.00000 (0.00000) | 0.00000 (0.00000) | 0.00000 (0.00000) | 0.00008 (0.00000) |

Values in brackets are Monte Carlo standard errors
